# Cooperative multivalent receptor binding promotes exposure of the SARS-CoV-2 fusion machinery core

**DOI:** 10.1038/s41467-022-28654-5

**Published:** 2022-02-22

**Authors:** Alexander J. Pak, Alvin Yu, Zunlong Ke, John A. G. Briggs, Gregory A. Voth

**Affiliations:** 1 Department of Chemistry, The University of Chicago, Chicago, IL USA; 2 Structural Studies Division, Medical Research Council Laboratory of Molecular Biology, Cambridge, UK; 3 Chicago Center for Theoretical Chemistry, The University of Chicago, Chicago, IL USA; 4 Institute for Biophysical Dynamics, The University of Chicago, Chicago, IL USA; 5 James Franck Institute, The University of Chicago, Chicago, IL USA; 6 Present Address: Department of Chemical and Biological Engineering, Colorado School of Mines, Golden, CO USA; 7 Present Address: Department of Cell and Virus Structure, Max Planck Institute of Biochemistry, Martinsried, Germany

**Keywords:** Computational biophysics, Single-molecule biophysics, Viral infection

## Abstract

The molecular events that permit the spike glycoprotein of severe acute respiratory syndrome coronavirus 2 (SARS-CoV-2) to bind and enter cells are important to understand for both fundamental and therapeutic reasons. Spike proteins consist of S1 and S2 domains, which recognize angiotensin-converting enzyme 2 (ACE2) receptors and contain the viral fusion machinery, respectively. Ostensibly, the binding of spike trimers to ACE2 receptors promotes dissociation of the S1 domains and exposure of the fusion machinery, although the molecular details of this process have yet to be observed. We report the development of bottom-up coarse-grained (CG) models consistent with cryo-electron tomography data, and the use of CG molecular dynamics simulations to investigate viral binding and S2 core exposure. We show that spike trimers cooperatively bind to multiple ACE2 dimers at virion-cell interfaces in a manner distinct from binding between soluble proteins, which processively induces S1 dissociation. We also simulate possible variant behavior using perturbed CG models, and find that ACE2-induced S1 dissociation is primarily sensitive to conformational state populations and the extent of S1/S2 cleavage, rather than ACE2 binding affinity. These simulations reveal an important concerted interaction between spike trimers and ACE2 dimers that primes the virus for membrane fusion and entry.

## Abstract (web summary)

Simulations reveal concerted interactions between the SARS-CoV-2 spike trimers and ACE2 receptors that result in cooperative spike binding and shedding, and further suggest that variant efficacy is promoted by increased RBD opening or S1/S2 cleavage efficiency.

## Introduction

Infection by severe acute respiratory syndrome coronavirus 2 (SARS-CoV-2) begins with the binding of viral particles to angiotensin-converting enzyme 2 (ACE2) cell-surface receptors, followed by viral-host cell membrane fusion, and entry of the viral genetic cargo^1^. These events are orchestrated by spike glycoproteins—trimeric class I fusion proteins—that decorate the exterior of SARS-CoV-2 particles. Each spike protein consists of S1 and S2 domains, which can be post-translationally cleaved by furin, and to a lesser extent by other proteases, and remain non-covalently associated in a metastable prefusion state, although the exact role of furin during infection is presently unclear^2–5^. The S1 domain contains the receptor binding domain (RBD) that is responsible for ACE2 recognition, while the S2 domain contains the fusion machinery. The S2 domains interact as a trimeric core with a characteristic helical stalk that is partially covered by the three S1 domains; the RBDs interact as a trimeric cap around the S2 trimeric core^6,7^. A hinge-like conformational change exposes the RBD interfaces that are amenable to ACE2 association. Receptor binding is believed to result in S1 shedding, thereby exposing the S2 core. For coronaviruses, it has been proposed that for fusion to occur, the second proteolytic site S2’ is first cleaved, thereby releasing the fusion peptide, after which the S2 core undergoes a dramatic conformational change into an extended helical stalk, i.e., the postfusion state^8^. If SARS-CoV-2 spike proteins undergo a similar sequence, then exposure of the S2 trimeric core is an important step prior to fusion.

As spike proteins are indispensable to SARS-CoV-2 infectivity, inhibiting their function is a natural target for therapeutic design. Understanding how spike proteins bind to ACE2 and how binding primes exposure of the S2 trimeric core is therefore essential to viral activity. Structures resolved using cryo-electron microscopy (cryo-EM) combined with docking have shown that ACE2-RBD binding requires spike trimers to be open, and that two spike trimers can be accommodated on one ACE2 dimer^9^. Alternatively, cryo-EM structures have shown that up to three soluble ACE2 monomers can bind to the same spike trimer, which may result in the exposure of the S2 trimeric core^10^; indeed, soluble ACE2 trimers were recently demonstrated as an effective inhibitor of SARS-CoV-2^11^. Both modalities are potentially important for viral avidity and require that the RBDs throughout the spike trimer stochastically open^6,7^, although it is unclear if RBDs open independently or cooperatively due to ACE2 interactions. To this end, the possible importance of the flexibility exhibited by both the stalk in membrane-bound spike trimers^12^ and ACE2 domains^13^ in aiding viral avidity is presently unknown. It has also been suggested that ACE2 binding to spike trimers induces allosteric effects that heighten conformational motions near proteolytic cleavage sites and the fusion peptide while dampening motions near the central helix and stalk^14^, or conformational motions that promote subsequent RBD opening^15^. Characterization of three circulating variant strains (Alpha [B.1.1.7], Beta [B.1.351], and Gamma [P.1]) has shown that increased infectivity is, in part, due to mutations in the RBD that increase ACE2 binding affinity^16,17^. Clearly, viral binding, fusion, and entry rely on a complex dynamical process that likely leverages the concerted interactions between spike trimers and ACE2 receptors.

To elucidate the molecular events that lead to binding and priming of the spike protein for membrane fusion, we use molecular dynamics (MD) simulations. Previous studies of virus-host avidity have suggested that microscopic events at cellular interfaces are potentially discrepant from those observed in systems with purified and soluble proteins, in part, due to the reduced dimensionality, hindered diffusion, and heterogeneous receptor and ligand concentrations in the former^18^. Our intention is to simulate the interface between spike-bound and ACE-bound membranes under controlled conditions and reveal possible cooperative microscopic events that emerge only at cellular interfaces. We first use atomistic MD simulations to systematically derive from “bottom-up” effective coarse-grained (CG) models of spike trimers and ACE2 dimers, which are then compared against conformational state statistics observed from experimental cryo-electron tomography (cryo-ET) data. We then use CG MD simulations to investigate the binding between membrane-bound spike trimers and membrane-bound ACE2 receptors, and associated shedding of the S1 domains that expose the S2 trimeric cores. While a prior cryo-EM model showed that spike ectodomains may bind up to three soluble ACE2^10^, our simulations show that up to two ACE2 dimers bind to a single spike trimer in a distinct counter-clockwise (from a top-down view) pattern. Our analysis indicates that binding between spike trimers and ACE2 dimers is positively cooperative, and furthermore, shows that this multivalent interaction is necessary to efficiently shed S1, a necessary step prior to membrane fusion. Finally, we investigate molecular factors that have been hypothesized to explain the increased infectivity of SARS-CoV-2 variants. Our findings show that productive dissociation of S1 by ACE2 is largely mediated by an increased propensity for multi-RBD opening and for S1/S2 cleavage, suggesting possible mechanisms for increased variant infectivity. At present, such insights are difficult to obtain from experiments alone^18^.

## Results

### Coarse-grained models of membrane-embedded spike trimers and ACE2 dimers

**Fig. 1:**
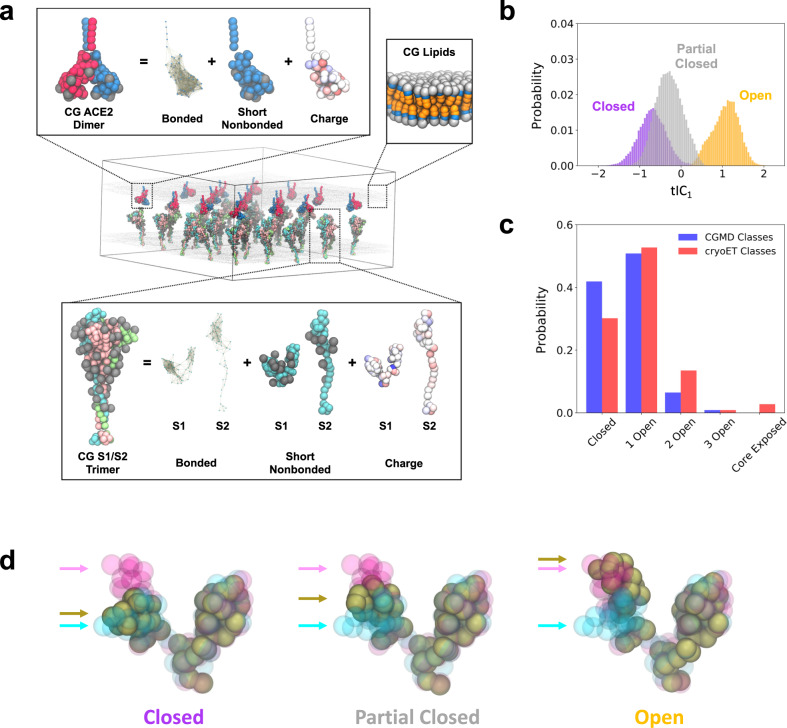
Schematic overview and characterization of our coarse-grained (CG) models. **a** Representative depiction of a CG simulation of spike trimers in membrane interacting with an adjacent membrane with ACE2 dimers. The insets depict the CG model components for the spike trimer (bottom), ACE2 dimer (upper left), and lipid membrane (upper right). Note that the protein CG sites are colored by monomer while glycans are represented by gray balls. **b** Probability distributions for RBD conformations projected onto the first time-lagged independent component (tIC_1_). The purple, gray, and orange distributions (*N* = 19383, 33810, and 21810, respectively) denote the three clusters identified by k-means clustering with labels indicating their representative conformational state. **c** Comparison of spike trimer conformational state populations (ranging from 0 to 3 open RBDs or shed RBDs, i.e., exposure of the S2 trimeric core) from CG simulations (*N* = 53076) and cryo-ET classification (*N* = 4220); the core exposed state is in the prefusion form in the CG simulations while it is in the postfusion form in the cryo-ET dataset. **d** Representative depiction of the average configuration of CG S1 (brown beads) within the identified k-means clusters. For reference, CG configurations of experimentally-resolved open and closed states of S1 are shown as magenta and cyan beads, respectively. Arrows indicate the positions of each respective RBDs. In all cases, the N-terminal domains of S1 are aligned.

Our CG MD simulations consist of three constituents, spike trimers, ACE2 dimers, and lipid membranes, each depicted in Fig. 1a. We extended our previously reported implicit-solvent CG spike protein model derived from all-atom MD trajectories^19^, which were based on the open and closed state atomic models from cryo-EM (PDB 6VYB and 6VXX)^3^ that were further modified prior to atomistic MD simulations^20^. Each spike protomer is modeled as non-covalently interacting S1 and S2 domains with a CG resolution of around 10–15 amino acids per CG “bead” or site, and initially prepared as trimers; at this resolution, collective conformational motions and interactions between subgroups of residues are retained while detailed information on interactions between individual residues is lost. Previous biochemical data has indicated that a subpopulation of uncleaved spike (S0) is also present on virions, although their relative fraction widely varies while their distribution across trimers is unclear^3,4,6,7,21–29^. Here, for simplicity, we initially model our trimers with 100% cleavage at the S1/S2 boundary. In select cases, we also investigate the impact of partial cleavage at the S1/S2 boundary. Briefly, intra-domain interactions are represented by a heterogeneous effective harmonic bond network while inter-domain interactions are represented by a combination of excluded volume repulsion, short-range attraction, and screened electrostatic terms (see “Methods”). A hybrid model capable of sampling both open and closed states was constructed through linear combination of the open and closed CG model Hamiltonians with equal weight. A similar resolution and interaction model are used to represent each ACE2 monomer, initially prepared as dimers; we note that the Zn(II) ion bound to the peptidase domain^30^ is implicitly represented in the CG model and is assumed to remain bound. The lipids are represented by a phenomenological CG model with a bending rigidity of 30 *k*_*B*_*T* to emulate the mechanical properties of typical membranes^31^. All CG model details and parametrization procedures are described in the “Methods” section.

It is well-known that the RBDs of spike trimers are capable of hinge-like movements, resulting in combinations of open and closed configurational states throughout the trimer^2,3,6,7^. Therefore, to validate our updated CG spike model, we quantified the configurational sampling of the RBD. We first simulated a single CG spike trimer in membrane (see “Methods”) for 50 × 10^6^ CG timesteps (*τ*_CG_ = 50 fs). To characterize the motions of the RBD, we quantified the distribution of reciprocal interatomic distances^32^ (DRID) for each configuration of the three RBDs; the DRID method computes the first, second, and third moments of the reciprocal distances between each CG site and every other CG site, yielding 3*N*_CG_ features. We next performed dimensional reduction using time-lagged independent component analysis (tICA), a linear projection technique that aims to maximize representation of auto-correlations (see Supplementary Fig. 1)^33^. The first 10 tIC dimensions were clustered using k-means clustering^34^, revealing three primary conformational states. These three states can be seen in Fig. 1b, which depicts the probability distribution of each of the three labeled clusters along the first tIC (tIC_1_) dimension. Assessing the root-mean-squared deviation of the configurations from each of the three states (see Fig. 1d and Supplementary Fig. 2) reveals that the purple-labeled distribution represents “closed” RBDs, the orange-labeled distribution represents “open” RBDs, and the gray-labeled distribution represents an intermediate state we denote as “partially closed,” which we discuss later. The cumulative probabilities of the closed, open, and partially closed states are 0.26, 0.29, and 0.45, respectively. The separability of the open- and closed-state distributions (we include the partially-closed state in the latter) depicted in Fig. 1b suggests that tIC_1_ can be used as a classifying metric for the open state (tIC_1_ > 0.4) and closed or partially-closed states (tIC_1_ < 0.4).

In our prior cryo-ET classification of RBDs within spike trimers along the surface of intact virions, we found that RBD conformations are stochastic and may range from three closed RBDs to three open RBDs, with three closed RBDs and one open (two closed) RBDs representing the two most predominant states^7^. Given these observations, we assessed the distribution of open RBDs within spike trimers as predicted by our CG model. We prepared 25 spike trimers in a flat membrane (around 170 × 170 nm^2^) with periodic boundary conditions and simulated three replicas of the system for 172 × 10^6^ *τ*_CG_. The simulated surface density, which is around 1150 nm^2^ per spike trimer, was chosen to reflect virions observed using cryo-ET, which had 24 ± 9 spike trimers and an average diameter of 91 nm^7^. Using the final 12.5 × 10^6^ *τ*_CG_, we characterized the RBDs throughout each of the trimers and computed the population of trimers with 0, 1, 2, and 3 open RBDs. In Fig. 1c, we compare the distribution of trimer states from our CG simulations to that of our previously reported cryo-ET dataset^7^. Our CG spike trimers undergo a slow yet reversible transition between open and closed states, with 51% of the total population having 1 open RBD, 42% having 0 open RBDs (i.e., 3 closed), and diminishing populations of 2 and 3 open RBD trimers (6 and 1%, respectively). We note that the relative populations of observed states are also sensitive to sample purification and fixation procedures, as demonstrated by other cryo-ET studies of spike trimer conformations in virions. For instance, Yao et al. identified 54% closed trimers from the prefusion population while Turoňová et al. identified 36% closed trimers with the remaining population identified as 1 open RBD (19%), an intermediate between closed and 1 open RBD (21%), and a third unidentified state (24%)^7,12^. Our present model favors the 1 open RBD state, but is also adjustable to favor other states, as we demonstrate later. Note that complete S2 trimeric core exposure (i.e., the precursor to the postfusion state) was not observed throughout these simulations. However, we observed partial exposure in rare cases, i.e., single S1 domains from spike trimers spontaneously dissociated due to thermal fluctuations, which we discuss further below. In contrast, a minority population of postfusion trimers were previously observed by cryo-ET. At this point, it is unclear whether this discrepancy is due to limitations of the CG model, due to fixation by formaldehyde which may shift the open/closed equilibrium, or both. Nonetheless, our analysis provides qualitative validation that the conformational variability of the RBD in our CG spike trimer is consistent with experimental observations.

### Spike trimers can bind to multiple ACE2 dimers

To investigate the spike-ACE2 binding process, we simulated spike trimers interfacing with ACE2 dimers. We prepared triplicate simulations of spike trimers in one membrane adjacent to another containing ACE2 dimers (see Fig. 1a). The density of spike trimers and ACE2 dimers was fixed such that the relative ratio of ACE2 dimer to spike trimer ([ACE2_dimer_]/[*S*_trimer_]) was 2.56, i.e., 64 ACE2 dimers and 25 spike trimers, sufficient to allow multivalent ACE2 binding to each spike trimer. The two membranes (and their constituents) were initially shifted along their normal directions until the closest distance between spike trimers and ACE2 dimers was around 2 nm. The simulations were run for 172 × 10^6^ *τ*_CG_.

Throughout our simulations, we observed spike trimers fluctuating between the open and closed state, with the former preceding ACE2 binding, as expected. Interestingly, we observed spike trimers that were capable of binding to multiple ACE2 dimers, which required the additional opening of RBDs. However, we did not see multiple spike trimers binding to the same ACE2 dimer, suggesting that any multivalent interactions between spike and ACE2 are due to an excess of accessible ACE2 receptors.

Our simulations predict two modalities for ACE2-facilitated dissociation of S1. First, S1 dissociation appears to occur due to thermal fluctuations, which may happen both in the absence and presence of binding between an open RBD and a single ACE2 dimer. The remaining two S1 monomers persist on the spike trimer, although in some cases, complete S1 shedding occurs after secondary RBD opening and binding to a new ACE2 receptor. The second modality was observed more frequently and involves the sequential binding of two ACE2 receptors to the same spike trimer. Consequently, dissociation of all three S1 domains is accelerated and results in the exposure of the S2 trimeric core (see Supplementary Movie 1).

**Fig. 2:**
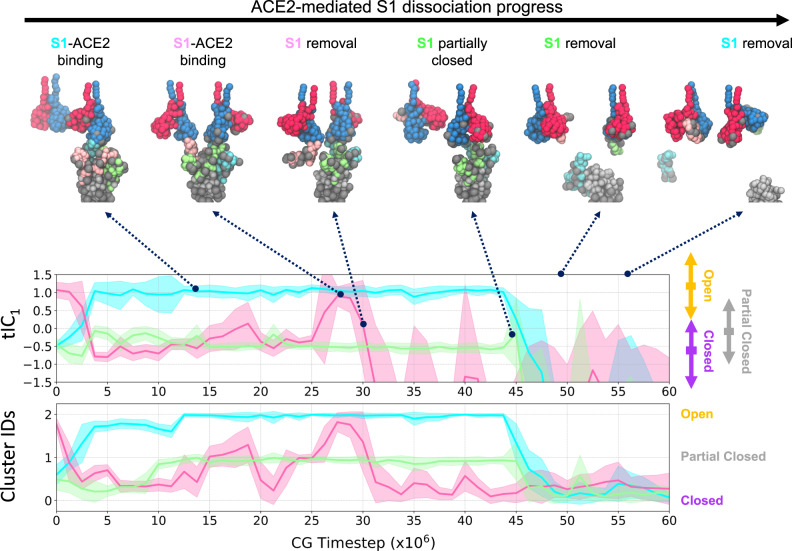
Dissociation of S1 facilitated by multivalent ACE2 binding. (Top) Snapshots of one representative S1 dissociation process upon binding of two ACE2 dimers. Each monomer in the ACE2 dimer is represented by red and blue beads, respectively, while each S1 protomer in the spike trimer is represented by cyan, pink, and green beads, respectively. The gray and silver beads denote glycans and S2 protomers, respectively. The depicted process is also shown in Supplementary Movie 1. (Middle) Time series profile depicting tIC_1_ evaluated for the RBD of each protomer (sharing the same color) for the spike trimer depicted above, i.e., spike trimer protomers are labeled cyan to pink to green (back to cyan) in counter-clockwise order when viewing from the top-down. To the right of the panel, the double-ended arrows represent the extent of the tIC_1_ distributions for each state from Fig. 1b, while the rectangle shows the peak of each distribution. (Bottom) Time series profile depicting k-means cluster IDs for the RBD of each protomer (same color scheme as the middle panel). To the right of the panel, the structural class assigned to each cluster ID is annotated. Each time series profile depicts the mean (line) and standard deviation (shaded region) of *N* = 25000 points using block averaging over 500 points.

We depict the representative elements of the multivalent ACE2-binding process in Fig. 2, in addition to tIC_1_ time-series profiles for each RBD (additional examples are shown in Supplementary Fig. 3). Here, the observed effect on the bound spike trimer is one of processive release of S1 domains in a cyclical fashion. The process generally adheres to the following sequential steps. First, RBDs conformationally vary between the open and closed state, as seen for *τ* < 5 × 10^6^ *τ*_CG_ (note the variation of the cyan and pink lines/protomers in Fig. 2). One ACE2 dimer successfully binds to the open RBD, pulling the RBD and enforcing the open state, as seen for *τ* = 12 × 10^6^ CG (the cyan protomer adopts tIC_1_ = 1.0 with reduced variance). The cavity formed between the upraised RBD and the N-terminal arm of S1 provides a stabilizing pocket for the clockwise RBD (from a top-down view), or the green protomer as depicted in Fig. 2 (with −0.6 < tIC_1_ < −0.2), thereby “tightening” the adhesion of this S1 domain; here, the green protomer adopts the partially closed state. Conversely, the counter-clockwise RBD, or the pink protomer as depicted in Fig. 2, appears to “loosen” its adhesion to the same cavity provided by the green S1 domain. Due to the weakened interactions, the pink protomer dynamically switches between the closed and partially-closed state, as seen between 10 × 10^6^ < τ < 25 × 10^6^ *τ*_CG_ (pink line/protomer in Fig. 2 with −0.75 < tIC_1_ < 0.0). Thermal fluctuations allow the pink protomer to spontaneously adopt the open state (*τ* = 26 × 10^6^ *τ*_CG_). A second ACE2 dimer binds to the newly exposed RBD and “pulls” this S1 domain away from the spike, thereby promoting dissociation (*τ* = 30 × 10^6^ *τ*_CG_). The remaining S1 dimer complex, which remains bound to the first ACE2 dimer, subsequently “loosens” and eventually dissociates due to thermal fluctuations (*τ* = 45 × 10^6^ *τ*_CG_). Finally, the S1-bound ACE2 dimers diffuse away from the spike trimer, which now consists of the exposed S2 trimeric core (*τ* > 50 × 10^6^ *τ*_CG_).

### Structural changes to S1 upon ACE2 binding

**Fig. 3:**
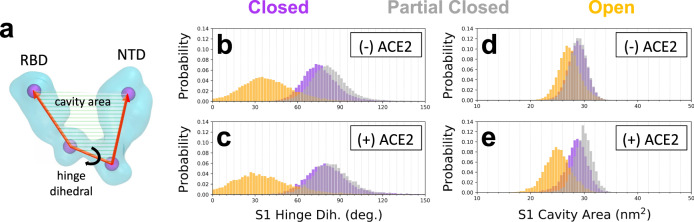
Structural changes to S1 upon ACE2 binding. **a** Schematic of the four geometric points (purple spheres) in S1 used to define the hinge dihedral and the cavity area. **b**, **c** Probability distributions of the hinge dihedral in each of the three structural states (closed, partially closed, and open) in the absence (*N* = 75003) and presence (*N* = 43800) of ACE2. **d**, **e** Probability distributions of the cavity area in each of the three structural states (closed, partially closed, and open) in the absence (*N* = 75003) and presence (*N* = 43800) of ACE2.

We further analyze structural differences in S1 upon ACE2 binding in Fig. 3. Here, we show the probability distribution of two geometric factors (Fig. 3a), the dihedral angle describing the hinge-like motion of the RBD and the area between the four points that define the dihedral angle, which we use as a proxy for the area of the cavity, i.e., the area occupied by the RBD of the clockwise protomer. In the open state, the peak of the dihedral angle distribution shifts downward by 7° (Fig. 3b, c) upon ACE2 binding while the peak of the cavity area distribution shifts downward by 2 nm^2^ (Fig. 3d, e), indicating that the cavity that may be occupied by the clockwise RBD reduces in volume upon ACE2 binding. If the clockwise RBD is within this cavity, the constriction due to ACE2 binding may impede its ability to escape. Interestingly, in the partially closed state, ACE2 binding shifts the peak of the cavity area distribution upward by 1 nm^2^ (Fig. 3d, e), and the expansion of this cavity may facilitate RBD release. On the basis of the events described in Fig. 2, we propose that ACE2 binding restricts the clockwise S1 into the partially closed state, which subsequently facilitates RBD opening of the counter-clockwise S1. We also analyze the pairwise distance distributions between close contacts at the intra-protomer and inter-protomer S1/S2 interfaces (Supplementary Fig. 4). We find that inter-protomer contacts successively diminish as S1 transitions from the closed to ACE2-bound open state, while intra-protomer contacts remain comparable, thereby suggesting that S1 dissociation is due to weakened inter-protomer interactions.

### Cooperativity between spike trimers and ACE2 dimers

Prior experimental binding assays using soluble ACE2 have demonstrated positive cooperativity when binding to SARS-CoV-2 spike trimers^35^. Our simulations show that multivalent ACE2 interactions induce productive S1 dissociation, and further suggest that exposure of the postfusion machinery may be cooperatively correlated with ACE2 expression. To test this hypothesis, we prepared additional triplicate simulations with the same spike trimer density but varied the ACE2 density. Here, we tested stoichiometric ratios of [ACE2_dimer_]/[*S*_trimer_] between 0 and 4, the latter representing 100 ACE2 dimers and 25 spike trimers, and performed simulations for 172 × 10^6^ *τ*_CG_. The final 12.5 × 10^6^ *τ*_CG_ were used for analysis.

**Fig. 4:**
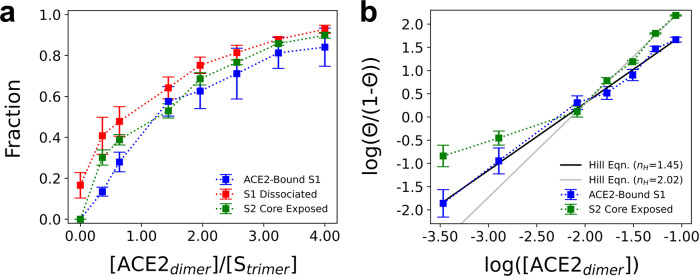
Cooperative enhancement of S2 trimeric core exposure by ACE2. **a** Summary statistics for the fraction of S1 monomers bound to ACE2 (blue), S1 monomers dissociated from the spike trimer (red), and spike trimers with complete exposure of the S2 trimeric core (green) as the stoichiometric ratio of ACE2 dimers to spike trimers, [ACE2_dimer_]/[*S*_trimer_], is varied. **b** Hill plot comparing the natural log of the surface density of ACE2 (in #/nm^2^) to the natural log of the fraction of S1 monomers bound to ACE2 or the fraction of completely exposed S2 trimeric cores (Θ). The black and gray lines are fits to the Hill equation with Hill coefficients (*n*_H_) equal to 1.45 and 2.02. All data points report the mean and standard error from triplicate simulations.

We summarize statistics on the extent of ACE2 binding, S1 dissociation, and S2 trimeric core exposure as [ACE2_dimer_]/[*S*_trimer_] is varied in Fig. 4a. We used a distance-based metric (described in “Methods”) to determine both binding and dissociation events. First, we find that S1 monomers are capable of spontaneous dissociation due to thermal fluctuations in the absence of ACE2, as noted above. Within our simulated timescales, however, none of these dissociation events resulted in complete S2 trimeric core exposure. As S1 dissociation was not observed in our atomistic MD simulations, nor during training of the CG model, we speculate that the collective membrane fluctuations, further induced by the spike trimer inclusions, promote S1 dissociation as bound S1 should be considered a metastable state.

The presence of ACE2 has intriguing effects on binding, dissociation, and exposure statistics. To measure the degree of cooperativity, we fit our binding data to the Hill equation:

1 $${{{{{\rm{ln}}}}}}\frac{\Theta }{1-\Theta }={n}_{{{{{\rm{H}}}}}}{{{{{\rm{ln}}}}}}\left[A\right]+c$$

where Θ is the fraction of S1 monomers bound to ACE2 (or fraction of completely exposed S2 trimeric cores), [*A*] is the surface density of ACE2, *n*_H_ is the Hill coefficient, and *c* is a constant. As seen in Fig. 4b, we find that *n*_H_ > 1 (= 1.45) for the binding curve, indicating that S1 binding to ACE2 is positively cooperative. In Fig. 4a, both the extent of S1 dissociation and S2 trimeric core exposure appear to monotonically increase with [ACE2_dimer_]/[*S*_trimer_]. Note that the relative ratio between S1 dissociation and S2 core exposure significantly decreases when [ACE2_dimer_]/[*S*_trimer_] ratios exceed 2. In fact, as seen in Fig. 4b, the propensity for S2 core exposure when [ACE2_dimer_]/[*S*_trimer_] > 2 also exhibits positive cooperativity with *n*_H_ = 2.02. Together, these results suggest that multivalent ACE2 binding promotes S1 dissociation events that productively expose the S2 trimeric core. We note that the postfusion machinery requires a substantial conformational change once S2 is exposed^8^, which is outside of the scope of our current study.

### Impact of variant emulating mutations on spike trimer binding and dissociation

**Fig. 5:**
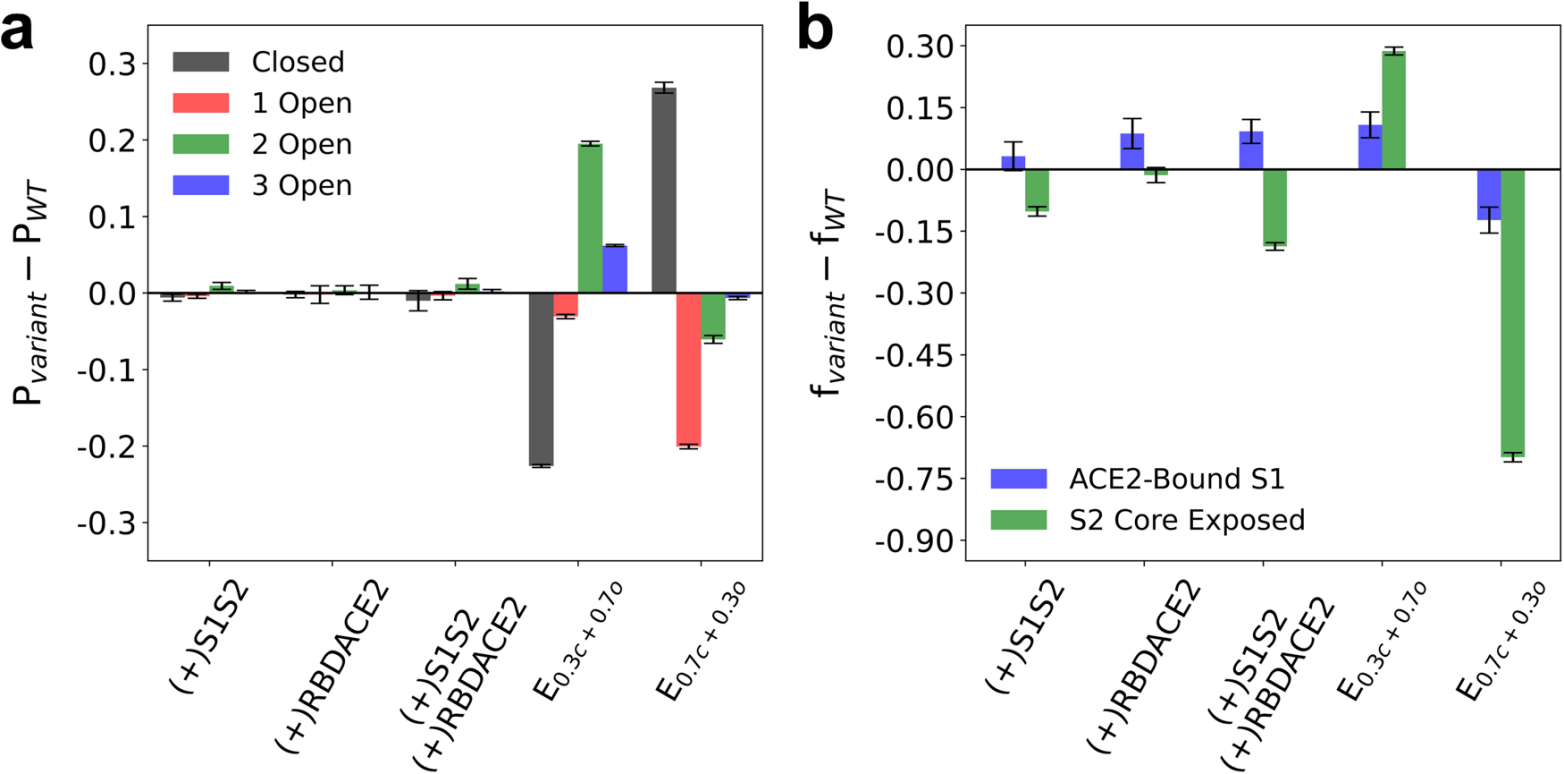
Comparison of variant-emulating CG models to that of the wild type (WT). **a** Summary statistics for the difference in the probabilities of the closed (black), 1 RBD open (red), 2 RBD open (green), and 3 RBD open (blue) conformational states for the variants and that of the WT (*P*_variant_ − *P*_WT_). **b** Summary statistics for the difference in the fraction of S1 monomers bound to ACE2 (blue) and spike trimers with complete exposure of the S2 trimeric core (green) for the variants and that of the WT (*f*_variant_ – *f*_WT_); here, the stoichiometric ratio of ACE2 dimers to spike trimers is 2.56:1 (i.e., 64 ACE2 dimers to 25 spike trimers). The five variant-emulating CG models are: enhanced S1–S2 interactions ((+)S1S2), enhanced RBD-ACE2 interactions ((+)RBDACE2), enhanced S1–S2 and RBD-ACE2 interactions ((+)S1S2(+)RBDACE2), increased open-state conformational sampling (E_0.3c+0.7o_), and increased closed-state conformational sampling (E_0.7c+0.3o_). All data points report the mean and standard error from triplicate simulations.

We next investigated the impact of biophysical factors recently described for SARS-CoV-2 variants on spike trimer binding and dissociation. For instance, the D614G mutation is more infectious than the original (WT) virus, which has been ascribed to stabilized interactions between S1 and S2 that prevent dissociation^36^, promote RBD opening^37,38^, or increase ACE2 affinity^39^. The latter two factors have also been observed in other variants, including the Alpha, Beta, and Gamma variants^29,40,41^. We prepared five variant-emulating CG models by perturbing the attractive terms in the CG Hamiltonian (see “Methods”). We enhanced attractive interactions at the intra-protomer S1/S2 interface, which we denote the (+)S1S2 model. We similarly enhanced the interactions at the RBD/ACE2 interface, i.e., the (+)RBDACE2 model. The third model consisted of enhanced interactions at both the S1/S2 and RBD/ACE2 interfaces, i.e., the (+)S1S2(+)RBDACE2 model. As indicated by Fig. 5a, these three models have negligible impact on the conformational states (e.g., closed or open) explored by the CG spike trimer. The fourth and fifth models were constructed by adjusting the linear mixing coefficients of the closed and open CG model Hamiltonians. The fourth and fifth models consisted of a respective 30%/70 and 70%/30% blend of the closed and open states, i.e., the E_0.3c+0.7o_ and E_0.7c+0.3o_ models. As seen in Fig. 5a, the E_0.3c+0.7o_ model promotes the population of 2- and 3-RBD open states by 19.5 ± 0.6 and 6.1 ± 0.2%, respectively, while the E_0.7c+0.3o_ model promotes the population of the closed state by 26.8 ± 1.2%. We emphasize that these models are not representative of any particular variant, but instead characteristic of general variant behavior.

For each variant-emulating CG model, using the same simulation procedure described above, we quantified the extent to which S1 monomers bound to ACE2 and S2 trimeric cores exposed upon binding. In Fig. 5b, we depict the difference between the fraction of S1 bound and S2 core exposed in each variant-emulating model to that of the WT (refer to Fig. 4a); here, we focus only on [ACE2_dimer_]/[*S*_trimer_] = 2.56, which is beyond the onset of cooperative ACE2-induced S1 binding and S2 exposure. Interestingly, we find that increasing the strength of RBD/ACE2 interactions increases the efficiency of S1 binding to ACE2 by 8.6 ± 3.6%, yet appears to have no significant effect on S2 trimeric core exposure. On the other hand, increasing the strength of S1/S2 interactions decreases the efficiency of S2 trimeric core exposure by 10.2 ± 1.1%, which is to be expected. The most notable impact to S2 trimeric core exposure is observed when the population of open and closed states is perturbed. While increasing the propensity for remaining closed (E_0.7c+0.3o_) impedes the efficiency of S2 trimeric core exposure by 69.9 ± 1.1%, increasing the propensity for 2- and 3-RBD open states (E_0.3c+0.7o_) facilitates S2 trimeric core exposure efficiency by 28.7 ± 0.9%. Our results suggest that rather than the energetics of interactions at the RBD/ACE2 and S1/S2 interfaces, the relative population of the RBD open to closed states dictates the exposure efficacy of the S2 trimeric core.

**Fig. 6:**
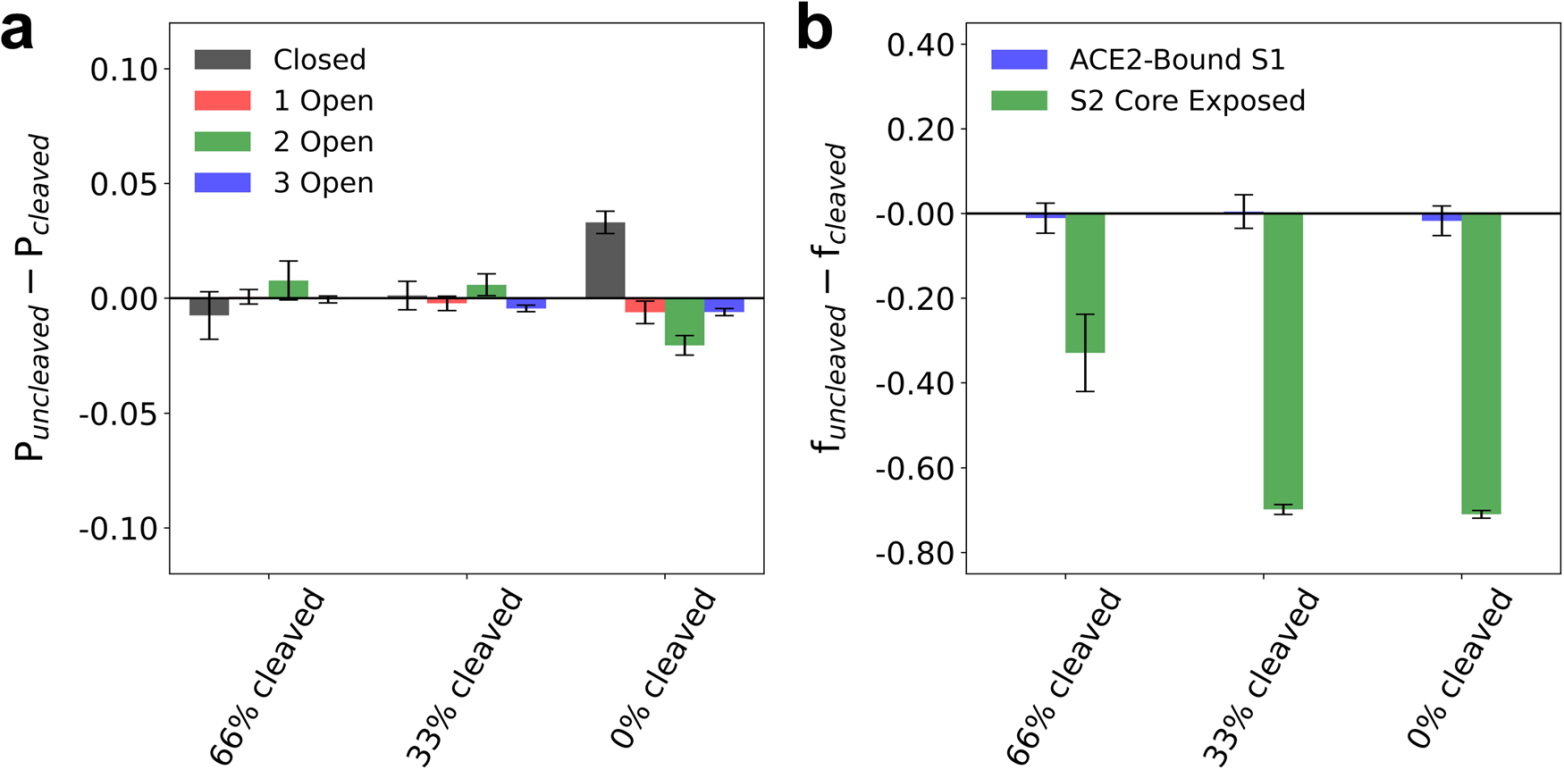
Comparison between spike trimer S1/S2 cleavage states for the wild type (WT) CG model. **a** Summary statistics for the difference in the probabilities of the closed (black), 1 RBD open (red), 2 RBD open (green), and 3 RBD open (blue) conformational states for the partially cleaved and completely cleaved spike trimer states (*P*_uncleaved_ − *P*_cleaved_) for WT virus. **b** Summary statistics for the difference in the fraction of S1 monomers bound to ACE2 (blue) and spike trimers with complete exposure of the S2 trimeric core (green) for the partially cleaved and completely cleaved spike trimer states (*f*_uncleaved_ − *f*_cleaved_) for WT virus; here, the stoichiometric ratio of ACE2 dimers to spike trimers is 2.56:1 (i.e., 64 ACE2 dimers to 25 spike trimers). In each simulation, the cleavage states are homogeneously distributed, e.g., the 66% cleaved system consists of 2 out of 3 cleaved protomers in every spike trimer. All data points report the mean and standard error from triplicate simulations.

To this point, we have modeled spike trimers with complete cleavage at the S1/S2 boundary. Yet as mentioned above, western blot assays have reported a wide distribution of S0 (i.e., uncleaved) protomer populations, ranging from 0 to 60% with no clear dependence on cell line or expression method^3,4,6,7,21–25^. The S1/S2 cleavage site consists of a polybasic arginine motif (R682-R685; RRAR) that attenuates cleavage upon mutation^4,24,42^. The P681H and P681R mutations, expressed in the Alpha/Omicron [B.1.1.529] and Kappa [B.1.617.1]/Delta [B.1.617.2] variants, respectively, may increase S1/S2 cleavage efficiency^27,43^, although contradictory reports suggest that these mutations have negligible impact on cleavage^25,26,28^. Nonetheless, as these variants also exhibit increased infectivity compared to WT virus^44,45^, we sought to simulate the effects of partial S1/S2 cleavage within the spike trimer on ACE2 binding and S1 dissociation. We prepared additional CG model modifications in which the spike trimers consisted of varying fractions of cleaved and uncleaved spike protomers, ranging from three uncleaved protomers (i.e., 0% cleaved) up to one out of three uncleaved protomers (i.e., 66% cleaved). We performed the same computational assays as the ones described above for the variant-emulating CG models. As seen in Fig. 6a, partial cleavage of the spike trimer has no statistical effect on the populations of RBD open states. However, the uncleaved spike trimer exhibits slightly suppressed populations of RBD open states (at most, by 2.1 ± 0.7%) in comparison to that of the completely- and partially-cleaved spike trimers, while the population of the RBD closed state is slightly enhanced by 3.3 ± 0.8%.

The extent of S1/S2 cleavage throughout the spike trimer has a striking effect on S1 dissociation. As shown in Fig. 6b, both 0 and 33% cleaved trimers effectively prevent S1 dissociation and S2 trimeric core exposure; for comparison, recall that completely cleaved trimers at the same ACE2 surface density exhibit a 71.0 ± 0.9% propensity for S2 trimeric core exposure. In contrast, 66% cleaved trimers are amenable to S1 dissociation and S2 trimeric core exposure, although the efficiency of the latter is suppressed by 32.9 ± 9.1%. In all three cases, the propensity for S1-ACE2 binding is unaffected, suggesting that cooperative binding between S1 and ACE2 is independent of the S1/S2 cleavage state. However, it is clear that productive S2 trimeric core exposure requires at least two out of three protomers within the spike trimer to be cleaved, while the greatest efficacy is exhibited when all three protomers are cleaved.

On the basis of both our variant-emulating and partially-cleaved spike trimer CG simulations, we propose that SARS-CoV-2 variants with notably increased infectivity compared to the WT virus may be due to mutations that functionally increase the propensity for multiple open RBDs and decrease the population of uncleaved S0, thereby facilitating exposure of the S2 trimeric core, which contains the fusion machinery necessary for viral entry. As both S1-ACE2 binding and S1 dissociation are positively cooperative with respect to ACE2, we speculate that both functional factors enable variants to replicate at lower ACE2 expression levels compared to the WT virus.

## Discussion

We developed and applied CG simulation models of SARS-CoV-2 spike trimers and receptor ACE2 dimers to study the dynamical mechanisms involved in viral avidity and dissociation, the latter being a necessary step prior to membrane fusion. Our simulations recapitulate the metastability of the S1 domains, which remain bound to spike trimers after S1/S2 cleavage. Although rare, we observed spontaneous dissociation of S1 even in the absence of ACE2 binding, consistent with cryo-EM characterization of postfusion trimers on mature virions^6,7^. It has been shown that the D614G mutation is more infectious and stabilizes interactions between S1 and S2 that prevent dissociation^36^, promote RBD opening^37,38^, or increase ACE2 affinity^39^. Ostensibly, the D614G mutation prevents premature S1 dissociation and facilitates productive RBD-ACE2 binding, while ACE2-mediated S1 dissociation remains unimpeded. It would be valuable for future atomistic and CG simulations to ascertain how D614G and related mutations (e.g., the Alpha, Delta, and Omicron variants) affect spike trimer stability before, during, and after ACE2 binding.

One of our key findings is that the primary binding modality between spike trimers and ACE2 dimers involves multiple ACE2 dimers binding to single spike trimers, rather than multiple spike trimers binding to the same ACE2 dimer, an observation that is consistent with split reporter assays of soluble spike trimers incubated with ACE2 dimers^46^. We found that spike trimer binding exhibits positive cooperativity with ACE2 dimer density (an *n*_H_ of 1.45) due to the multivalent nature of ACE2 binding (see Fig. 4). Soluble ACE2 had previously been observed to cooperatively bind to SARS-CoV-2 spike trimers with *n*_H_ around 1.1–1.2, yet interestingly, not for SARS-CoV-1 spike trimers^35^. Finally, our simulations suggest that the multivalency of the ACE2 to spike trimer interaction is necessary to facilitate complete dissociation of the S1 monomers that occlude the S2 trimeric core, which contains the viral fusion machinery. Binding to ACE2 induces a cyclical tightening and loosening process that processively weakens S1 interactions, both to adjacent S1 monomers and to the underlying S2 core, which leads to S1 dissociation. On the basis of cryo-EM maps of soluble spike trimers bound to soluble ACE2, it was recently proposed that the three RBDs throughout the spike trimer open and bind to ACE2 in a processive fashion, eventually resulting in simultaneous dissociation of the three S1 monomers due to reduced contacts between neighboring monomers^10^. The process we observe in our simulations is conceptually similar, although we propose that only two ACE2 dimers are minimally necessary for productive uncoating of S1, and the counter-clockwise S1 (from a top-down view) from the initial ACE2-bound S1 tends to open, bind to a second ACE2, and dissociate first.

One notable difference between the conditions of our simulations and the experiments discussed above is that both our spike trimers and ACE2 dimers are tethered to their respective membranes in a more realistic system than solubilized proteins. The tethers inherently restrict the diffusion and conformational motions available to both spike and ACE2 proteins. One potential consequence is that the relative populations of different spike/ACE2 complexes for solubilized proteins may not reflect populations at the interface between virions and host cells. For instance, Benton et al. report a two-fold greater occurrence of the second RBD opening in the clockwise direction compared to the counter-clockwise direction after the first open RBD is bound to soluble ACE2^10^. During our CG simulations, however, the restraints on the S1 bound to the first ACE2 (see Fig. 3b, c) instead appear to widen the interior cavity of the clockwise S1, thereby releasing the RBD of the counter-clockwise S1. The S1 protein can be imagined as an “arm” with the clockwise RBD placed near its “elbow.” Upon binding to ACE2, the S1 protein “squeezes” due to stochastic pulling forces as both the spike and ACE2 proteins are tethered (see Fig. 3d, e). As a result, the clockwise RBD is restrained in the partially closed state (see Fig. 2), thereby preventing clockwise RBD opening. This clockwise S1 arm also “relaxes” (see Fig. 3d, e), which facilitates the closed-to-open transition of the counter-clockwise RBD. Likewise, these populations are likely dependent upon the relative stoichiometry of spike trimers and ACE2 dimers, as our simulations indicate. We suggest that the use of fluid membrane-bound spike trimers and ACE2 receptors in biochemical and structural characterization of viral avidity, e.g., as seen in the work of Yang et al. and Lu et al.^47,48^, compared to assays using soluble components, may prove to be characteristically different. For example, single-molecule Förster resonance energy transfer analysis suggests that spike trimers bound to one or two ACE2 receptors exhibit asymmetry throughout the three spike protomers^48^, which is consistent with the dynamical behavior observed in our CG simulations.

Our simulations of variant-emulating CG models suggest that one important function of variant mutations may be to increase the population of RBD open states, especially states with multiple open RBDs, rather than enhancing RBD binding affinity to ACE2 in order to facilitate ACE2-induced S1 dissociation (see Fig. 5). The originally identified virus is known to primarily favor the closed and 1 open RBD states^7^. Several cryo-EM studies of Alpha recently reported that the spike trimer exhibited comparatively increased open RBD state populations, ranging from 65 to 82% in the 1 open RBD state^29,40,41^. The remaining state populations were either in the closed or 2 open RBD state; intriguingly, Yang et al. reported that the 2 open RBD state accounted for 27% of the total population^41^. A similar series of cryo-EM studies on Kappa and Delta suggests that their spike trimers exhibit an increased propensity for RBD opening compared to WT virus, but potentially to a lesser extent than Alpha^28,49^. Interestingly, the Beta and Gamma variants appear to greatly favor RBD open states^28,29,40,49^, and possibly more so than the Alpha, Kappa, and Delta variants. Given that Delta is the dominant variant at present, while Alpha was formerly a dominant variant, an additional function due to variant mutations is likely to be important. Our partially-cleaved trimer simulations (see Fig. 6) suggest that the spike trimer must have, at minimum, two out of three protomers cleaved at the S1/S2 site in order for the S2 trimeric core to be exposed, while complete S1/S2 cleavage throughout the trimer greatly increases S2 trimeric core exposure efficiency. Both Beta and Gamma variants lack mutations near the S1/S2 cleavage site, while Alpha and Delta respectively have the P681H and P681R mutations, which may increase S1/S2 cleavage efficiency^27,43^. Hence in the context of currently available biochemical data, our analysis suggests that mutations that increase the propensity for both RBD opening and S1/S2 cleavage are essential aspects of dominant variants that have increased infectivity compared to the WT. The Omicron variant, which at present is rapidly spreading, has the P681H and N679K mutations, and it would be worthwhile to experimentally quantify both its S1/S2 cleavage efficiency and its population of RBD open states. Although outside the scope of this work, we also expect S1 mutations that aid neutralization escape to be another important function exhibited by dominant variants^17,50^.

Our analysis motivates several directions for future simulations. While researchers have explored the free energy surface of single RBD open-close transitions^51,52^, the thermodynamics of secondary and tertiary RBD opening, as well as the role of multivalent ACE2 binding, should be thoroughly investigated. As our analysis of variant-emulating CG models indicates that increased propensity for RBD opening facilitates S1 dissociation and S2 trimeric core exposure, it would be worthwhile to compare the thermodynamics of RBD opening across variants; our partially-cleaved trimer simulations suggest that the S1/S2 cleavage stage has a minor impact on RBD opening. We note that our current CG model will also likely require additional features to holistically probe SARS-CoV-2 virion binding, fusion, and entry. Here, we investigated spike trimer and ACE2 dimer association at the interface of two planar membranes. However, it will be important to elucidate the effects of virion curvature (between 70 and 120 nm diameters^7^), which likely impose additional restrictions to spike trimer conformational sampling while seeking ACE2 receptors. Sophisticated modeling approaches to incorporate large-scale conformational changes, such as the extension of the heptad repeat coiled-coil in the postfusion state^8^, will also be necessary. Structural information that continues to emerge should also be incorporated into the model, such as structural studies into the roles of dynamic regions such as the fusion-peptide proximal region (FPPR) that has been hypothesized to impede RBD opening, thereby stabilizing the closed state^6,53^. In this work, we linearly mixed the open- and closed-state Hamiltonians in order to adjust the population of configurational states. However, it would be interesting to investigate the conformational fidelity of CG models derived from newer structural models. Several additional approaches, including plastic network models^54^ and multi-configurational coarse-graining^55^, may prove useful to construct CG models capable of state transitions with increased accuracy for state populations and transition rates. These efforts will expand the capabilities of CG modeling, which in turn will provide valuable insight into collective molecular phenomena during SARS-CoV-2 infection, such as the role of cooperative multivalent ACE2 binding to induce spike trimer uncoating reported herein.

## Methods

### Atomistic MD simulations

Trajectories from prior atomistic MD simulations of the spike trimer in membrane were used to derive effective CG Hamiltonians^19,20^. Statistics for the open and closed state of the spike trimer were supplemented with additional triplicate simulations of the spike ectodomain using atomic models prepared previously in ref. ^17^. Triplicate simulations of the ACE2 dimer interacting with bound RBDs were also prepared using an initial structure from PDB 6M17 (ref. ^9^). All simulations were prepared using CHARMM-GUI^56^ and the CHARMM36m^57^ force field. Each system was solvated with 2 nm of TIP3P water from the protein edge to the simulation domain boundary and with 150 mM NaCl. All simulations were performed using GROMACS 2020^58^. Minimization was performed using steepest descent until the maximum force was reduced to 1000 kJ/mol/nm^2^. Then, equilibration was performed in several phases. First, 10 ns were integrated in the constant *NVT* ensemble using the stochastic velocity rescaling thermostat^59^ with a damping time of 0.2 ps and a timestep of 1 fs. During this phase, the Cα backbone of the protein was harmonically restrained with a force constant of 1000 kJ/mol/nm^2^. An additional 40 ns were integrated in the constant *NPT* ensemble using the stochastic velocity rescaling thermostat^59^ (2 ps damping time) and the Parrinello–Rahman barostat^60^ (10 ps damping time) and a timestep of 2 fs. Finally, an additional 1650 ns were integrated in the constant *NVT* ensemble using the Nose–Hoover chain thermostat^61^ (2 ps damping time) and a timestep of 2 fs. Throughout this procedure, H-containing bonds were constrained using the LINCS algorithm^62^. Statistics were gathered every 100 ps and the final 1200 ns of each trajectory was used for CG model generation.

### CG modeling

CG model generation for the protein constituents proceeded in three phases. The first phase determined the mapping between atomistic and CG sites. Mapping was performed using the essential dynamics coarse-graining (EDCG) method^63^ with resolutions chosen to balance between computational efficiency and least-squared error in the represented principal component subspace. Monomers of S1, S2, and ACE2 were mapped to 60, 50, and 70 CG sites, respectively; each N-linked glycan^64^ was mapped to a single site, totaling 13, 9, and 7 sites, respectively.

The second phase determined the effective CG Hamiltonian for each protein. We systematically determined parameters for S1/S2 in the open state, S1/S2 in the closed state, and ACE2 in the bound state, as well as their inter-domain interactions. The Hamiltonian (*E*_tot_) was decomposed into four terms: intra-domain fluctuations (*E*_intra_), inter-domain exclusion (*E*_excl_), inter-domain attraction (*E*_attr_), and screened electrostatics (*E*_yukawa_):

2 $${E}_{{{{{{\rm{tot}}}}}}}={E}_{{{{{{\rm{intra}}}}}}}+{E}_{{{{{{\rm{excl}}}}}}}+{E}_{{{{{{\rm{attr}}}}}}}+{E}_{{{{{{\rm{yukawa}}}}}}}$$

where *E*_intra_ was represented as a hetero-elastic network model (hENM) method^65^ with bond energies *k*(*r* − *r*_0_)^2^ where *k* is the spring constant of a particular bond and *r*_*0*_ is the equilibrium bond length. These parameters were optimized using the hENM method with a cutoff distance of 3, 4, and 3 nm for S1, S2, and ACE2, respectively. For *E*_excl_, a soft cosine potential, $A\left[1+{{{{{\rm{cos }}}}}}\frac{\pi {r}}{{r}_{c}}\right]$, was used, where *A* = 25 kcal/mol and *r*_*c*_ is the onset for excluded volume determined from pair correlation functions. For *E*_attr_, the sum of two Gaussian potentials, ${A}_{1}{{{{{\rm{exp }}}}}}\left[-\frac{{\left({r}_{{ij}}-{r}_{1}\right)}^{2}\,}{2{\sigma }_{1}^{2}}\right]+{A}_{2}{{{{{\rm{exp }}}}}}\left[-\frac{{\left({r}_{{ij}}-{r}_{2}\right)}^{2}\,}{2{\sigma }_{2}^{2}}\right]$, was used, where *r*_*1/2*_ and *σ*_*1/2*_ are the mean and standard deviation determined by a fit to the pair correlation between CG sites *i* and *j* through least-squares regression. The constants *A*_*1*_ and *A*_*2*_ were optimized using relative-entropy minimization (REM)^66^. For *E*_yukawa_, a Yukawa potential, $\frac{{q}_{i}{q}_{j}}{{4\pi \epsilon }_{r}{\epsilon }_{0}{r}_{{ij}}}{{{{{\rm{exp }}}}}}\left(-\kappa {r}_{{ij}}\right)$, was used, where *q*_*i*_ is the aggregate charge of CG site *i*, *κ* = 1.274 nm^−1^ is the inverse Debye length for 150 mM NaCl, and *ε*_*r*_ is the effective dielectric constant of the protein environment, approximated as 17.5^67^.

The third phase determined an effective Hamiltonian for the spike trimer that was capable of continuously transitioning between the open and closed state. Here, we modified *E*_intra_, *E*_excl_, and *E*_attr_ as follows. For *E*_intra_, we kept bonds that appeared in both open and closed states and used the mean *k* and *r*_*0*_ constants. For *E*_excl_, we used the minimum *r*_*c*_ from both open and closed states. For *E*_attr_, we tested different linear combinations of the Gaussians parametrized from the open and closed state. We found that an equal mix recapitulated open/closed statistics the best, i.e., *E*_attr_ = 0.5*E*_attr,open_ + 0.5*E*_attr,closed_. No modification of *E*_yukawa_ was needed since this term is not state-dependent.

The variant-emulating CG spike models described above were constructed by either (i) applying different scalar multipliers to the prefactors for specific *E*_attr_ terms (for the (+)S1S2, (+)RBDACE2, and (+)S1S2(+)RBDACE2 models) or (ii) adjusting the coefficients in the *E*_attr,open_ and *E*_attr,closed_ summation (for the E_0.3c+0.7o_ and E_0.7c+0.3o_ models; the former consists of 30% *E*_attr,closed_ and 70% *E*_attr,open_ while the latter is the reverse). For the former, a complete list of multipliers for each involved pairwise interaction is provided in Supplementary Table 4. The uncleaved CG spike models described above were constructed using an additional ENM between S1 CG types 58–60 and S2 CG types 74–76, which are the sites located at the S1/S2 boundary; a spring constant of 1 kcal/mol/nm^2^ was used.

The lipid model used the functional form from ref. ^31^. with tuned parameters listed in the Supplementary Information. For the purposes of modeling the interaction between the transmembrane domains of the spike trimer/ACE2 dimer and lipids, we effectively considered the transmembrane domain as having the same interaction as the lipids. Complete details on protein and lipid model parametrization are provided in Supplementary Information and Supplementary Figs. 5–7.

### CG simulations

All simulations were prepared using PACKMOL and Moltemplate^68,69^. Briefly, CG spike trimers and ACE2 dimers were arranged in a grid using each density described in the main text. Two CG lipid membranes were randomly packed around the transmembrane domains of each grid of proteins in a 200 × 200 nm^2^ lateral (*xy*) domain; the *z* dimension of the box was fixed to 160 nm. The two membranes were then translated along the *z* direction until the minimum *z* distance between spike trimers and ACE2 dimers was 2 nm. All simulations were performed using LAMMPS^70^. Conjugate gradient energy minimization was performed on each system until the change in force was less than 10^−6^. Then, proteins were held fixed while lipids were integrated using a Langevin thermostat at 310 K (0.5 ps damping constant) and a Berendsen barostat at 1 atm (5 ps damping constant, applied over the lateral *xy* dimension) over 2 × 10^6^ *τ*_CG_ (with *τ*_CG_ = 50 fs). During this process, the *xy* simulation domain compressed to around 170 × 170 nm^2^. We then integrated both proteins and lipids using a Langevin thermostat at 310 K (5 ps damping constant) over 170 × 10^6^ *τ*_CG_. During the first 5 × 10^6^ *τ*_CG_, all attractive interactions between CG spike trimers and ACE2 dimers were deactivated to allow random diffusion without influence from possible spike/ACE2 interactions. Trajectory snapshots were saved every 25 × 10^3^ *τ*_CG_.

### Analysis

All analysis was performed using a combination of VMD^71^ scripts and Python scripts using the MSMBuilder^72^ and MDTraj^73^ libraries. The configurational state of each RBD was featurized using the DRID method^32^. Dimensional reduction (via tICA^33^) of this feature set into 10 tICs was performed using a lag time of 40 × 10^3^ *τ*_CG_; the values of the first tIC eigenvector are shown in Supplementary Fig. 1. The data were then clustered into 3 sets using k-means clustering^34^. We featurized and projected the configuration of each RBD extracted from our CG simulations onto the first tIC to classify its state. We also used the following distance metric to classify the bound state of the S1 domain to both its partner S2 domain and ACE2:

3 $$\left|\frac{1}{{N}_{{ii}}}\mathop{\sum }\limits_{{ii}}^{{N}_{{ii}}}{r}_{{{{{{\rm{S1}}}}}},{ii}}-\frac{1}{{N}_{{jj}}}\mathop{\sum }\limits_{{jj}}^{{N}_{{jj}}}{r}_{{{{{{\rm{S2}}}}}}\left({{{{{\rm{ACE}}}}}}2\right),{jj}}\right|={d}_{{{{{{\rm{S1-S2}}}}}}({{{{{\rm{ACE}}}}}}2)}$$

where *ii* and *jj* refer to CG site indices out of a set with *N*_*ii*_ and *N*_*jj*_ total sites, *r* is the position, *d* is the distance, and || is the norm of the vector. In other words, we compute the distance between the centers of geometry of sets of CG sites. We chose CG site clusters that were observed to have close association distances with minimal fluctuation during CG model training. For S1/S2 binding, these included site indices 31–32 and 54–57 for S1 and 77, 87–89, 98–99, and 102 for S2. For S1/ACE2 binding, these included site indices 43–49 for S1 and 134–137, 158, and 162 for ACE2 (residue mapping for each site is shown in Supplementary Tables 1–3). Using this metric, we classified S1/S2 and S1/ACE2 as bound states when *d*_S1–S2_ and *d*_S1-ACE2_ were less than 4 nm, respectively.

To define the S1 dihedral angle and cavity area, we used reference points constructed from the center of geometry of four groups of CG sites: (i) CG site types 33, 51, 52, 53; (ii) CG site types 42–49; (iii) CG site types 31–32, 54, 58–60; (iv) CG site types 9, 11, 13–17, 26–27.

### Reporting summary

Further information on research design is available in the Nature Research Reporting Summary linked to this article.

## Supplementary information

Supplementary Information

Description of Additional Supplementary Files

Supplementary Movie 1

Reporting Summary

## Data Availability

The data that support this study are available from the corresponding author upon reasonable request. All relevant data and models generated from this study are deposited to Zenodo [https://zenodo.org/record/5919230]. Atomic structures used as reference throughout this study are available from the PDB under accession codes 6VYB, 6VXX, and 6M17.

## Code availability

Model parameters, input files, and custom code are available on Github [https://github.com/uchicago-voth/MSCG-models/tree/master/Spike_ACE2] and Zenodo [https://zenodo.org/record/5919230].

## References

[1] Shang J (2020). Cell entry mechanisms of SARS-CoV-2. Proc. Natl Acad. Sci. USA.

[2] Wrapp D (2020). Cryo-EM structure of the 2019-nCoV spike in the prefusion conformation. Science.

[3] Walls AC (2020). Structure, function, and antigenicity of the SARS-CoV-2 spike glycoprotein. Cell.

[4] Papa G (2021). Furin cleavage of SARS-CoV-2 Spike promotes but is not essential for infection and cell–cell fusion. PLOS Pathog..

[5] Hoffmann M, Kleine-Weber H, Pohlmann S (2020). A multibasic cleavage site in the spike protein of SARS-CoV-2 is essential for infection of human lung cells. Mol. Cell.

[6] Cai Y (2020). Distinct conformational states of SARS-CoV-2 spike protein. Science.

[7] Ke Z (2020). Structures and distributions of SARS-CoV-2 spike proteins on intact virions. Nature.

[8] Walls AC (2017). Tectonic conformational changes of a coronavirus spike glycoprotein promote membrane fusion. Proc. Natl Acad. Sci. USA.

[9] Yan R (2020). Structural basis for the recognition of SARS-CoV-2 by full-length human ACE2. Science.

[10] Benton DJ (2020). Receptor binding and priming of the spike protein of SARS-CoV-2 for membrane fusion. Nature.

[11] Xiao T (2021). A trimeric human angiotensin-converting enzyme 2 as an anti-SARS-CoV-2 agent. Nat. Struct. Mol. Biol..

[12] Turoňová B (2020). In situ structural analysis of SARS-CoV-2 spike reveals flexibility mediated by three hinges. Science.

[13] Barros, E. P. et al. The flexibility of ACE2 in the context of SARS-CoV-2 infection. *Biophys. J.* **120**, 1072–1084 (2021). 10.1016/j.bpj.2020.10.036 PMC7661960 33189680

[14] Raghuvamsi PV (2021). SARS-CoV-2 S protein:ACE2 interaction reveals novel allosteric targets. eLife.

[15] Xu C (2021). Conformational dynamics of SARS-CoV-2 trimeric spike glycoprotein in complex with receptor ACE2 revealed by cryo-EM. Sci. Adv..

[16] Zhou D (2021). Evidence of escape of SARS-CoV-2 variant B.1.351 from natural and vaccine-induced sera. Cell.

[17] Supasa P (2021). Reduced neutralization of SARS-CoV-2 B.1.1.7 variant by convalescent and vaccine sera. Cell.

[18] Erlendsson S, Teilum K (2021). Binding revisited—avidity in cellular function and signaling. Front. Mol. Biosci..

[19] Yu A (2020). A multiscale coarse-grained model of the SARS-CoV-2 virion. Biophys. J..

[20] Casalino L (2020). Beyond shielding: The roles of glycans in the SARS-CoV-2 spike protein. ACS Cent. Sci..

[21] Laporte M (2021). The SARS-CoV-2 and other human coronavirus spike proteins are fine-tuned towards temperature and proteases of the human airways. PLoS Pathog..

[22] Hoffmann M (2020). SARS-CoV-2 cell entry depends on ACE2 and TMPRSS2 and is blocked by a clinically proven protease inhibitor. Cell.

[23] Wrobel AG (2020). SARS-CoV-2 and bat RaTG13 spike glycoprotein structures inform on virus evolution and furin-cleavage effects. Nat. Struct. Mol. Biol..

[24] Johnson BA (2021). Loss of furin cleavage site attenuates SARS-CoV-2 pathogenesis. Nature.

[25] Lubinski B (2022). Functional evaluation of the P681H mutation on the proteolytic activation of the SARS-CoV-2 variant B.1.1.7 (Alpha) spike. iScience.

[26] Dicken, S. J. et al. Characterisation of B.1.1.7 and Pangolin coronavirus spike provides insights on the evolutionary trajectory of SARS-CoV-2. Preprint at *bioRxiv*10.1101/2021.03.22.436468 (2021).

[27] Brown, J. C. et al. Increased transmission of SARS-CoV-2 lineage B.1.1.7 (VOC 2020212/01) is not accounted for by a replicative advantage in primary airway cells or antibody escape. Preprint at *bioRxiv*10.1101/2021.02.24.432576 (2021).

[28] Zhang J (2021). Membrane fusion and immune evasion by the spike protein of SARS-CoV-2 Delta variant. Science.

[29] Gobeil Sophie, M. C. et al. Effect of natural mutations of SARS-CoV-2 on spike structure, conformation, and antigenicity. *Science* **373**, eabi6226. 10.1126/science.abi6226 PMC8611377 34168071

[30] Lubbe L, Cozier GE, Oosthuizen D, Acharya KR, Sturrock ED (2020). ACE2 and ACE: Structure-based insights into mechanism, regulation, and receptor recognition by SARS-CoV. Clin. Sci..

[31] Grime, J. M. A. & Madsen, J. J. Efficient simulation of tunable lipid assemblies across scales and resolutions. Preprint at https://arxiv.org/abs/1910.05362 (2019).

[32] Zhou T, Caflisch A (2012). Distribution of reciprocal of interatomic distances: A fast structural metric. J. Chem. Theory Comput..

[33] Naritomi Y, Fuchigami S (2011). Slow dynamics in protein fluctuations revealed by time-structure based independent component analysis: The case of domain motions. J. Chem. Phys..

[34] Lloyd S (1982). Least squares quantization in PCM. IEEE Trans. Inf. Theory.

[35] Anand, S. P. et al. Interaction of human ACE2 to membrane-bound SARS-CoV-1 and SARS-CoV-2 S glycoproteins. *Viruses* **12**, 1104 (2020). 10.3390/v12101104 PMC7601831 33003587

[36] Zhang L (2020). SARS-CoV-2 spike-protein D614G mutation increases virion spike density and infectivity. Nat. Commun..

[37] Mansbach RA (2021). The SARS-CoV-2 spike variant D614G favors an open conformational state. Sci. Adv..

[38] Benton DJ (2021). The effect of the D614G substitution on the structure of the spike glycoprotein of SARS-CoV-2. Proc. Natl Acad. Sci. USA.

[39] Ozono S (2021). SARS-CoV-2 D614G spike mutation increases entry efficiency with enhanced ACE2-binding affinity. Nat. Commun..

[40] Cai Y (2021). Structural basis for enhanced infectivity and immune evasion of SARS-CoV-2 variants. Science.

[41] Yang T-J (2021). Effect of SARS-CoV-2 B.1.1.7 mutations on spike protein structure and function. Nat. Struct. Mol. Biol..

[42] Sasaki M (2021). SARS-CoV-2 variants with mutations at the S1/S2 cleavage site are generated in vitro during propagation in TMPRSS2-deficient cells. PLoS Pathog..

[43] Peacock, T. P. et al. The SARS-CoV-2 variants associated with infections in India, B.1.617, show enhanced spike cleavage by furin. Preprint at *bioRxiv*10.1101/2021.05.28.446163 (2021).

[44] Davies NG (2021). Estimated transmissibility and impact of SARS-CoV-2 lineage B.1.1.7 in England. Science.

[45] Campbell, F. et al. Increased transmissibility and global spread of SARS-CoV-2 variants of concern as at June 2021. *Eurosurveillance* **26**, 2100509 (2021). 10.2807/1560-7917.ES.2021.26.24.2100509 PMC8212592 34142653

[46] Lui, I. et al. Trimeric SARS-CoV-2 Spike interacts with dimeric ACE2 with limited intra-Spike avidity. Preprint at *bioRxiv*10.1101/2020.05.21.109157 (2020).

[47] Yang J (2020). Molecular interaction and inhibition of SARS-CoV-2 binding to the ACE2 receptor. Nat. Commun..

[48] Lu M (2020). Real-Time Conformational Dynamics of SARS-CoV-2 Spikes on Virus Particles. Cell Host Microbe.

[49] Yang, T.-J. et al. Structure-activity relationships of B.1.617 and other SARS-CoV-2 spike variants. Preprint at *bioRxiv*10.1101/2021.09.12.459978 (2021).

[50] Liu C (2021). Reduced neutralization of SARS-CoV-2 B.1.617 by vaccine and convalescent serum. Cell.

[51] Gur M (2020). Conformational transition of SARS-CoV-2 spike glycoprotein between its closed and open states. J. Chem. Phys..

[52] Casalino, L. et al. AI-driven multiscale simulations illuminate mechanisms of SARS-CoV-2 spike dynamics. *Int. J. High Perform. Comput. Appl.*10.1177/10943420211006452 (2021). 10.1177/10943420211006452 PMC8064023 38603008

[53] Xiong X (2020). A thermostable, closed SARS-CoV-2 spike protein trimer. Nat. Struct. Mol. Biol..

[54] Maragakis P, Karplus M (2005). Large amplitude conformational change in proteins explored with a plastic network model: Adenylate kinase. J. Mol. Biol..

[55] Sharp ME, Vázquez FX, Wagner JW, Dannenhoffer-Lafage T, Voth GA (2019). Multiconfigurational coarse-grained molecular dynamics. J. Chem. Theory Comput..

[56] Jo S, Kim T, Iyer VG, Im W (2008). CHARMM-GUI: A web-based graphical user interface for CHARMM. J. Comput. Chem..

[57] Huang J (2016). CHARMM36m: An improved force field for folded and intrinsically disordered proteins. Nat. Methods.

[58] Abraham MJ (2015). GROMACS: High performance molecular simulations through multi-level parallelism from laptops to supercomputers. SoftwareX.

[59] Bussi G, Zykova-Timan T, Parrinello M (2009). Isothermal-isobaric molecular dynamics using stochastic velocity rescaling. J. Chem. Phys..

[60] Parrinello M, Rahman A (1980). Crystal structure and pair potentials: A molecular-dynamics study. Phys. Rev. Lett..

[61] Martyna GJ, Klein ML, Tuckerman M (1992). Nosé–Hoover chains: The canonical ensemble via continuous dynamics. J. Chem. Phys..

[62] Hess B, Bekker H, Berendsen HJC, Fraaije JGEM (1997). LINCS: A linear constraint solver for molecular simulations. J. Comput. Chem..

[63] Zhang Z (2008). A systematic methodology for defining coarse-grained sites in large biomolecules. Biophys. J..

[64] Watanabe Y, Allen JD, Wrapp D, McLellan JS, Crispin M (2020). Site-specific glycan analysis of the SARS-CoV-2 spike. Science.

[65] Lyman E, Pfaendtner J, Voth GA (2008). Systematic multiscale parameterization of heterogeneous elastic network models of proteins. Biophys. J..

[66] Shell MS (2008). The relative entropy is fundamental to multiscale and inverse thermodynamic problems. J. Chem. Phys..

[67] Li L, Li C, Zhang Z, Alexov E (2013). On the dielectric “constant” of proteins: Smooth dielectric function for macromolecular modeling and its implementation in DelPhi. J. Chem. Theory Comput..

[68] Martínez L, Andrade R, Birgin EG, Martínez JM (2009). PACKMOL: A package for building initial configurations for molecular dynamics simulations. J. Comput. Chem..

[69] Jewett AI, Zhuang Z, Shea J-E (2013). Moltemplate a coarse-grained model assembly tool. Biophys. J..

[70] Plimpton S (1995). Fast parallel algorithms for short-range molecular dynamics. J. Comput. Phys..

[71] Humphrey W, Dalke A, Schulten K (1996). VMD: Visual molecular dynamics. J. Mol. Graph..

[72] Beauchamp KA (2011). MSMBuilder2: Modeling conformational dynamics on the picosecond to millisecond scale. J. Chem. Theory Comput..

[73] McGibbon RT (2015). MDTraj: A modern open library for the analysis of molecular dynamics trajectories. Biophys. J..

