# Supplementary material for: Cooperative multivalent receptor binding promotes exposure of the SARS-CoV-2 fusion machinery core
Source: Nat Commun. 2022 Feb 22;13:1002. doi: 10.1038/s41467-022-28654-5 (PMC8863989; doi:10.1038/s41467-022-28654-5)
Supplement: Supplementary file 1 — Supplementary Information [file 41467_2022_28654_MOESM1_ESM.pdf]

Supplementary Information for

Cooperative multivalent receptor binding promotes exposure of the SARS-CoV-2 fusion machinery core

Alexander J. Pak[1,⊥], Alvin Yu[1], Zunlong Ke[5,#], John A. G. Briggs[5,#], and Gregory A. Voth[1,2,3,4,*]

[1] Department of Chemistry, The University of Chicago, Chicago, IL, USA
[2] Chicago Center for Theoretical Chemistry, The University of Chicago, Chicago, IL, USA
[3] Institute for Biophysical Dynamics, The University of Chicago, Chicago, IL, USA
[4] James Franck Institute, The University of Chicago, Chicago, IL, USA
[5] Structural Studies Division, Medical Research Council Laboratory of Molecular Biology, Cambridge, UK
[⊥] Present address: Department of Chemical and Biological Engineering, Colorado School of Mines, Golden, CO 80401
[#] Present address: Department of Cell and Virus Structure, Max Planck Institute of Biochemistry, Martinsried, Germany

* Corresponding author: gavoth@uchicago.edu

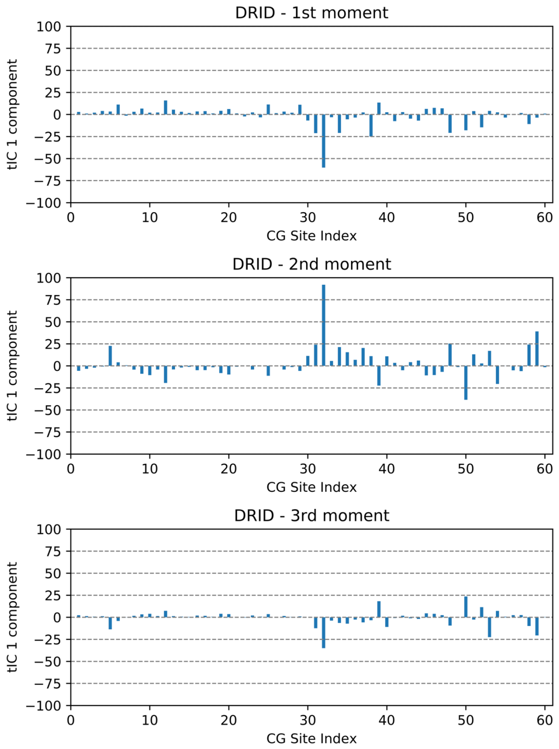

Supplementary Fig. 1. Eigenvector components of the 1st tIC with respect to the 1st through 3rd moments of DRID using the listed CG bead index within S1 as the reference point.

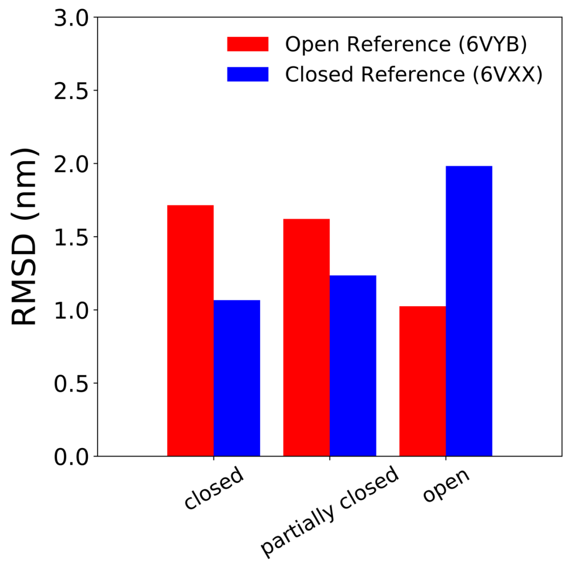

Supplementary Fig. 2. Root-mean-squared deviation (RMSD) of coarse-grained S1 configurations from the closed (N=19383), partially closed (N=33810), and open (N=21810) k-means clusters using the closed and open configurations from PDB 6VXX and 6VYB, respectively, as reference (1).

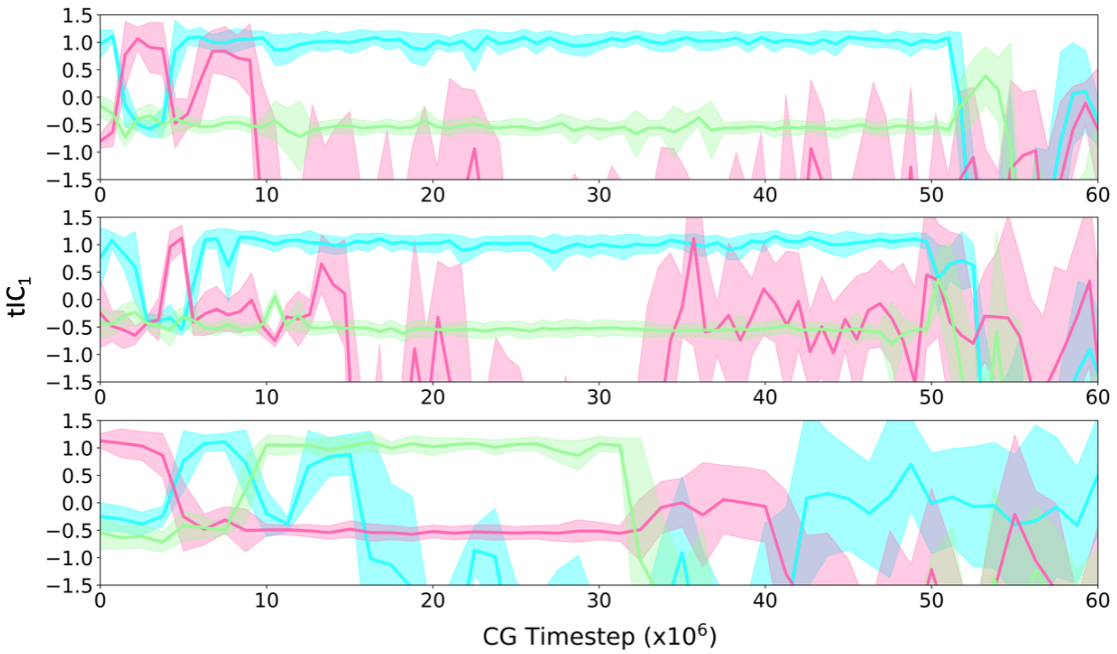

Supplementary Fig. 3. Additional time-series profiles of tIC_1 during ACE2-mediated S1 dissociation from spike trimers. Each of the three panels is an independent spike trimer. The colors are the same as **Fig. 2** in the main text. Note that the spike trimer protomers are labeled cyan to pink to green to cyan in counter-clockwise order when viewing from the top-down. These time series profiles indicate that the counter-clockwise protomer from the initially ACE2-bound protomer is bound next then dissociated. Each time series profile depicts the mean (line) and standard deviation (shaded region) of N=25000 points using block averaging over 500 points.

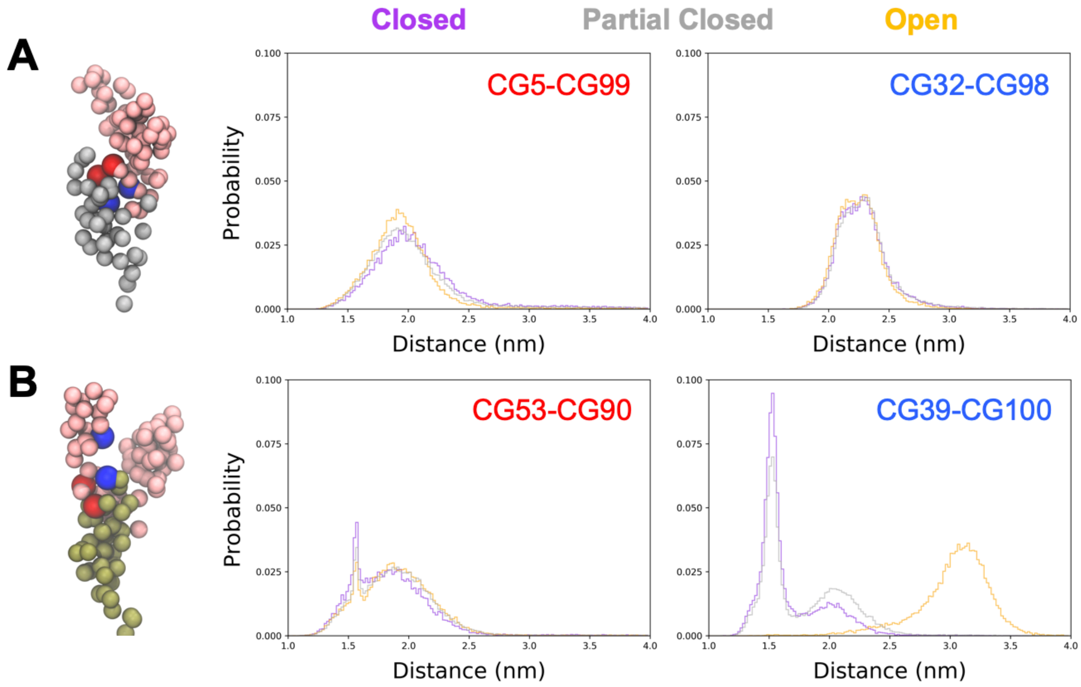

Supplementary Fig. 4. Probability distributions of pairwise distances between select CG sites at the (**A**) intra-protomer and (**B**) inter-protomer S1/S2 interface when S1 is in the closed (purple), partial closed (grey), and open (orange) states. The schematic depicts the S1 (pink balls), intra-protomer S2 (grey balls), and inter-protomer S2 in the clockwise direction (olive balls).

Supplementary Note: *Detailed description of coarse-grained modeling procedures*

Before coarse-grained (CG) model parameterization, each protein was mapped to CG beads using the essential dynamics coarse-graining (EDCG) method, which was designed to preserve the principal modes of motion sampled during atomistic simulations (2). In EDCG, the CG mapping operator, $\boldsymbol{M}_{\mathbf{R}}^{N}: \mathbf{r}^{n} \rightarrow \mathbf{R}^{N}$, which relates the configurations of the atomistic trajectory ($\mathbf{r}^{n}$) to that of the CG model ($\mathbf{R}^{N}$), is variationally optimized using simulated annealing. Here, each CG site is constructed using the center of mass of residues within contiguous segments of the protein's amino acid sequence. For N CG beads, $N-1$ segment boundaries throughout the amino acid sequence are adjusted to minimize the target residual

$$\chi^2 = \frac{1}{3N}\sum_{I=1}^{N} \langle \sum_{i,j} \left|\boldsymbol{r}_i - \boldsymbol{r}_j\right|^2 \rangle_t \quad : \; i,j \in I, j \geq i \tag{1}$$

where $I = 1, \ldots, N$ is the CG site index, the brackets $\langle \cdot \rangle_t$ denote a time-averaged quantity, the sum over i,j is a sum over all unique pairs in the set of atoms belonging to the CG site I, and $\boldsymbol{r}_i = \boldsymbol{x_i} - \langle \boldsymbol{x_i} \rangle_t$ is the displacement of atom i from its mean position $\langle \boldsymbol{x_i} \rangle_t$. Note that the residual is small when the displacements $\boldsymbol{r}_i$ and $\boldsymbol{r}_j$ are similar, i.e. the motions of atoms in the same CG site are correlated. We chose N using an "elbow" heuristic to identify when increasing N yielded diminishing returns in χ^2. The "elbow" region when comparing a range of N values with corresponding residual (i.e., χ^2) values, as depicted in **Fig. S5**, yields a CG mapping resolution that balances computational expense and mapping accuracy. Following this protocol, we used 60, 50, and 70 CG beads for the S1, S2, and ACE2 protein mappings, respectively. The N-linked glycans were removed prior to this procedure. Each glycan was mapped to its center of mass and appended to their corresponding EDCG-mapped protein.

Each CG model was described by intra-protein and inter-protein interactions, which were parameterized sequentially. Intra-protein interactions were represented using elastic network models (ENMs), which were designed to capture protein flexibility. We used the heterogeneous elastic network model (hENM) method, in which harmonic bonds are assigned to all pairs of CG particles within a tunable distance cutoff r_{cut} (3). The harmonic force constants are iteratively adjusted until the fluctuations in the CG model converge to that of the atomistic data, i.e.,

$$\frac{1}{k_{ij}^{n+1}} = \frac{1}{k_{ij}^{n}} - \alpha\left(\langle r_{ij}^2 \rangle_{\mathrm{CG}} - \langle r_{ij}^2 \rangle_{\mathrm{AA}}\right) \tag{2}$$

where k_{ij}^{n} is the harmonic force constant for each i,j CG pair at iteration n, $\langle r_{ij}^2 \rangle = \langle \left(x_{ij} - \langle x_{ij} \rangle\right)^2 \rangle$ is the mean-squared fluctuation for each i,j CG pair, and α is a parameter that controls the magnitude of the adjustment for each iteration. We chose r_{cut} for each CG model using the "elbow" heuristic as shown in **Fig. S6**. Here, our residual was $\sum_{i,j} \langle r_{ij}^2 \rangle_{\mathrm{CG}} - \langle r_{ij}^2 \rangle_{\mathrm{AA}}$. Following this protocol, we used distance cutoffs of 3, 4, and 3 nm for the S1, S2, and ACE2 CG models, respectively.

Inter-protein interactions were composed of excluded volume, attractive, and screened electrostatic terms. For excluded volume interactions, a phenomenological soft cosine potential was used

$$A\left[1+\cos\frac{\pi\, r}{r_c}\right] \tag{3}$$

where $A = 25\ \mathrm{kcal/mol}$ and r_c is the onset for excluded volume. For each CG pair, r_c was set to the minimum of two values: the minimum distance with non-zero frequency in the corresponding pairwise distance histogram from CG-mapped atomistic statistics or the default value of $r_c = 3.0\ \mathrm{nm}$. Screened electrostatics were modeled using Yukawa potentials

$$\frac{q_i q_j}{4\pi\epsilon_r\epsilon_0 r_{ij}}\exp(-\kappa\, r_{ij}) \tag{4}$$

where q_i is the aggregate charge of CG site i, $\kappa = 1.274\ \mathrm{nm}^{-1}$ is the inverse Debye length for 0.15 M NaCl, and ϵ_r is the effective dielectric constant of the protein environment, approximated as 17.5 (4).

Attractive non-bonded interactions between inter-protein contacts were modeled as the sum of two Gaussian potentials

$$A_1\exp\left[-\frac{\left(r_{ij}-r_1\right)^2}{2\sigma_1^2}\right]+A_2\exp\left[-\frac{\left(r_{ij}-r_2\right)^2}{2\sigma_2^2}\right] \tag{5}$$

where r_1 and σ_1 are the mean and standard deviation determined by a fit to the pair correlation functions (from CG-mapped atomistic statistics) between CG sites *i* and *j* through least-squares regression. The constants A_1 and A_2 were optimized using relative-entropy minimization (REM) (5). We used the iterative Newton-Raphson method(6) to update A_1 and A_2, which we refer to as parameter λ at iteration n

$$\lambda_{n+1} = \lambda_n - \chi\frac{\left(\frac{\partial S}{\partial\lambda}\right)}{\left(\frac{\partial^2 S}{\partial\lambda^2}\right)} \tag{6}$$

where χ is the “mixing ratio” or “learning rate” of the iterative optimization procedure. Each iteration required a LAMMPS simulation of a single copy of the CG protein using the current CG force field, which was run for 21×10^6 steps using a 100 fs timestep and a Langevin thermostat (10 ps damping time) at 300 K. Statistics were gathered every 800 steps over the final 20×10^6 steps to calculate the update shown in Eq. 6. To aid convergence during training, a learning rate schedule for χ was implemented:

1. $\chi = 0.5$ during the first 25 iterations
2. $\chi = 0.1$ during the next 75 iterations
3. $\chi = 0.01$ during the next 75 iterations
4. $\chi = 0.001$ during the next 75 iterations
5. $\chi = 0.1$ during the next 50 iterations

6. $\chi = 0.01$ during the final 150 iterations

A total of 450 iterations were used to generate each CG model. After this point, changes to λ were effectively zero, as seen in **Fig. S7**, and the optimization was considered complete.

The final component of our simulations, the phenomenological CG lipid model, followed the functional form presented in Ref. (7). We used a linear four-bead model with 1 head bead, 1 middle bead, and 2 tail beads. A piecewise potential was used to describe inter-lipid interactions

$$\begin{cases} -\left(\frac{2Ar_0}{\pi}\right)\sin\frac{\pi r}{2r_0}; \quad r \leq r_0 \\ -\left(\frac{B(r_c - r_0)}{\pi}\right)\sin\frac{\pi}{2} + \frac{(r - r_c)\pi}{r_c - r_0}; \quad r_0 < r < r_c \\ 0; \quad otherwise \end{cases} \tag{7}$$

where $r_0 = 1.2$ nm (or $r_0 = 0.9$ nm for the head bead), $r_c = 2.4$ nm, $B = 1.66$ k_BT, and $A = 50$ k_BT (20 k_BT for the head bead). Harmonic bonds and angles were used to describe intra-lipid interactions

$$K_b\left(r - r_{0,b}\right)^2 \tag{8}$$

$$K_a(\theta - \theta_0)^2 \tag{9}$$

where $K_b = 5$ k_BT/Å^2, $r_{0,b} = 0.6$ nm, $K_a = 5$ k_BT/rad^2, and $\theta_0 = \pi$ rad.

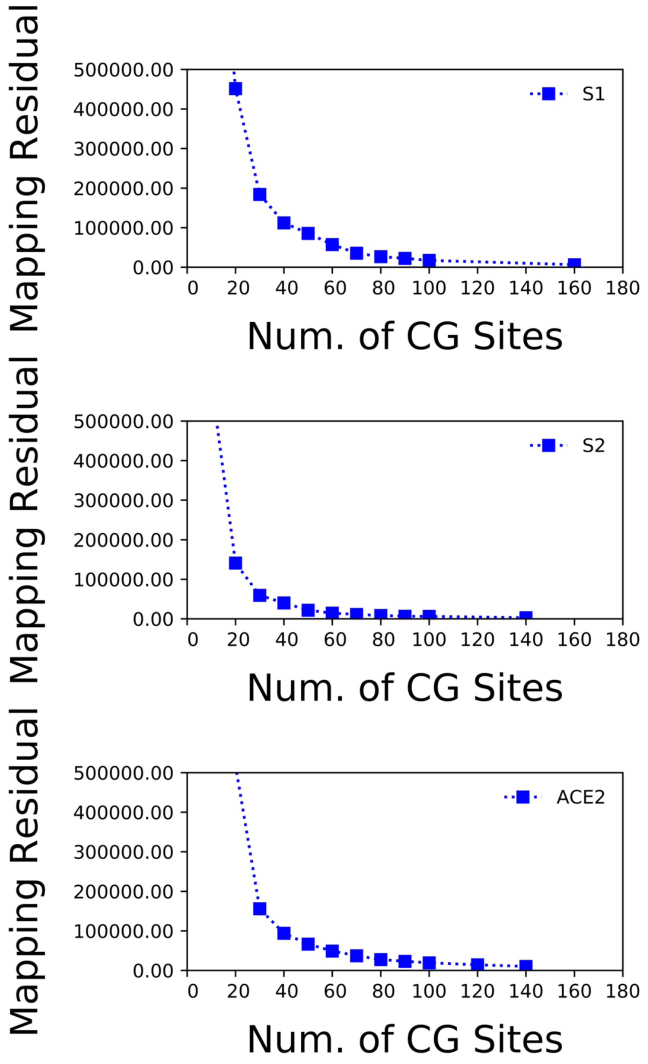

Supplementary Fig. 5. Comparison of EDCG residuals to CG model resolutions for each of the CG protein models: S1, S2, and ACE2.

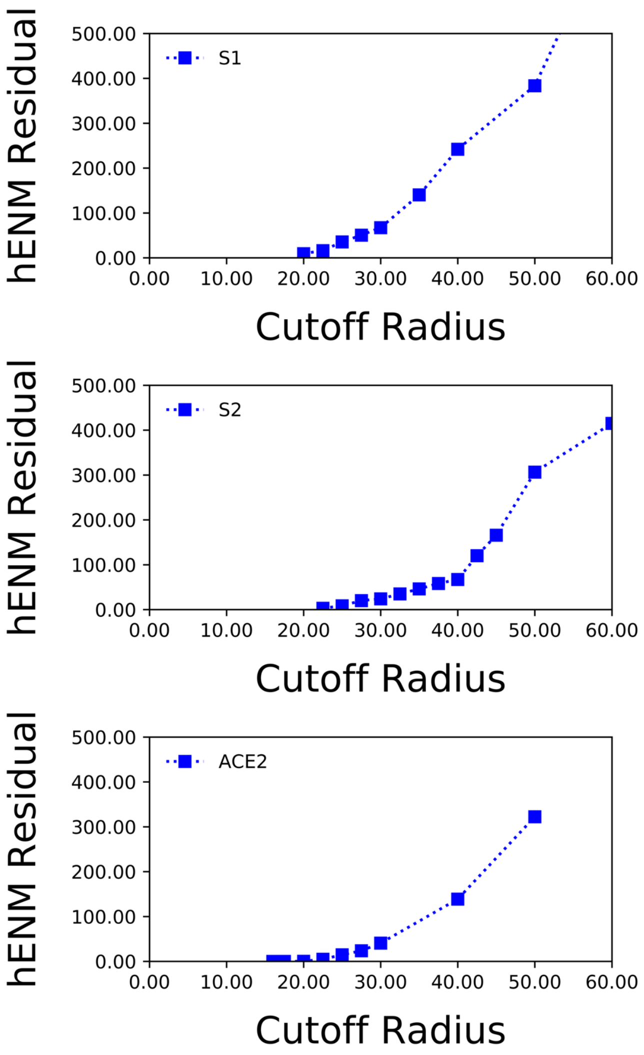

Supplementary Fig. 6. Comparison of hENM residuals to hENM cutoff radii for each of the CG protein models: S1, S2, and ACE2.

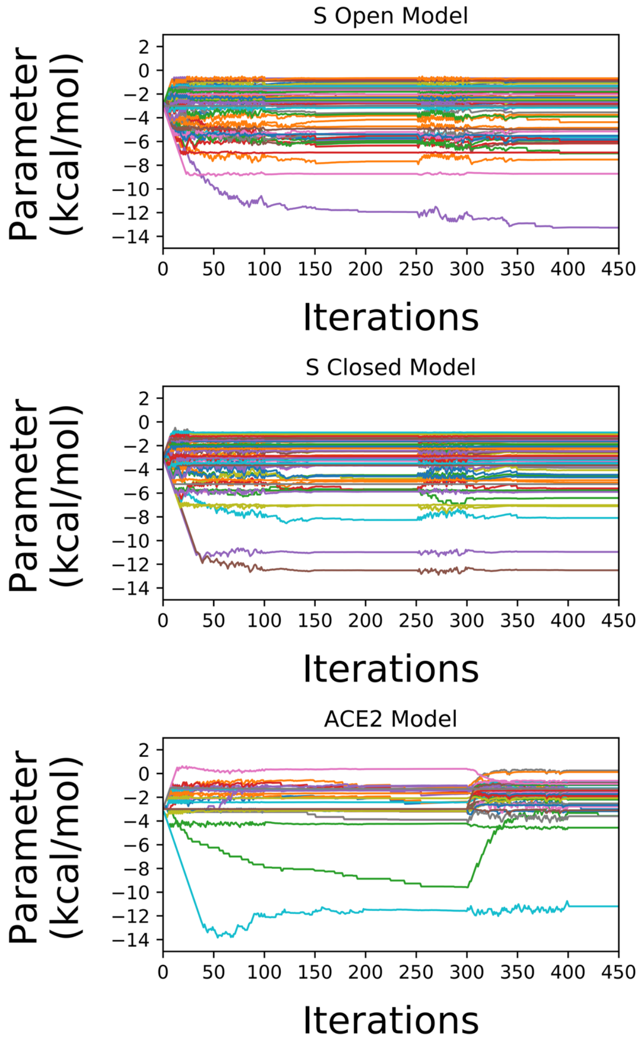

Supplementary Fig. 7. Comparison of Gaussian prefactor parameters as a function of REM optimization iteration number for each of the CG protein models: S (open state), S (closed state), and ACE2.

Supplementary Table 1. Summary of residue mapping to each coarse-grained site type in S1 (glycans (CG types 61-73) excluded).

S1 Protein

CG Type	Residue (Start)	Residue (End)	CG Index	Residue (Start)	Residue (End)
1	16	19	31	289	308
2	20	23	32	309	319
3	24	28	33	320	328
4	29	37	34	329	336
5	38	53	35	337	346
6	54	64	36	347	355
7	65	70	37	356	364
8	71	75	38	365	375
9	76	80	39	376	387
10	81	92	40	388	401
11	93	103	41	402	418
12	104	118	42	419	433
13	119	130	43	434	449
14	131	140	44	450	458
15	141	146	45	459	467
16	147	151	46	468	474
17	152	156	47	475	482
18	157	162	48	483	492
19	163	173	49	493	510
20	174	182	50	511	524
21	183	191	51	525	547
22	192	206	52	548	574
23	207	222	53	575	593
24	223	233	54	594	617
25	234	244	55	618	626
26	245	250	56	627	633
27	251	256	57	634	640
28	257	261	58	641	658
29	262	272	59	659	677
30	273	288	60	678	685

Supplementary Table 2. Summary of residue mapping to each coarse-grained site type in S2 (glycans (CG types 124-132) excluded).

S2 Protein

CG Type	Residue (Start)	Residue (End)	CG Index	Residue (Start)	Residue (End)
74	686	689	99	958	970
75	690	697	100	971	984
76	698	705	101	985	999
77	706	718	102	1000	1014
78	719	726	103	1015	1028
79	727	736	104	1029	1051
80	737	754	105	1052	1065
81	755	768	106	1066	1077
82	767	781	107	1078	1093
83	782	791	108	1094	1115
84	792	806	109	1116	1137
85	807	813	110	1138	1149
86	814	828	111	1150	1160
87	829	835	112	1161	1167
88	836	840	113	1168	1175
89	841	845	114	1176	1186
90	846	851	115	1187	1196
91	852	861	116	1197	1206
92	862	875	117	1207	1214
93	876	893	118	1215	1228
94	894	910	119	1229	1243
95	911	923	120	1244	1251
96	924	935	121	1252	1258
97	936	944	122	1259	1267
98	945	957	123	1268	1273

Supplementary Table 3. Summary of residue mapping to each coarse-grained site type in ACE2 (glycans (CG types 203-209) excluded).

ACE2 Protein

CG Type	Residue (Start)	Residue (End)	CG Index	Residue (Start)	Residue (End)
133	21	31	168	425	430
134	32	42	169	431	441
135	43	53	170	442	454
136	54	63	171	455	467
137	64	73	172	468	478
138	74	83	173	479	490
139	84	94	174	491	499
140	94	103	175	500	510
141	104	108	176	511	523
142	109	118	177	524	535
143	119	132	178	536	543
144	133	140	179	544	554
145	141	154	180	555	565
146	155	166	181	566	577
147	167	182	182	578	588
148	183	193	183	589	598
149	194	204	184	599	605
150	205	214	185	606	611
151	215	226	186	612	619
152	227	244	187	620	625
153	245	264	188	626	629
154	265	281	189	630	633
155	282	292	190	634	643
156	293	304	191	644	653
157	305	320	192	654	663
158	321	332	193	664	672
159	333	336	194	673	684
160	337	340	195	685	705
161	341	345	196	706	724
162	346	357	197	725	732
163	358	363	198	733	739
164	364	378	199	740	747
165	379	394	200	748	754
166	395	409	201	755	764
167	410	424	202	765	768

Supplementary Table 4. Summary of scalar multipliers applied to the prefactors A_1, A_2 in Eq. (5) for the listed CG type pairs for each variant-emulating CG model.

(+)S1S2

CG Type 1	CG Type 2	Multiplier
5	88	1.2
30	99	1.2
31	89	1.2
31	99	1.2
32	98	1.2

(+)RBDACE2

CG Type 1	CG Type 2	Multiplier
44	133	1.2
46	133	1.2
47	133	1.2
48	134	1.2
49	134	1.2
47	138	1.2
48	138	1.2
43	158	1.2
49	158	1.2
49	162	1.2
49	165	1.2

(+)S1S2(+)RBDACE2

CG Type 1	CG Type 2	Multiplier
5	88	1.2
30	99	1.2
31	89	1.2
31	99	1.2
32	98	1.2
44	133	1.2
46	133	1.2
47	133	1.2
48	134	1.2
49	134	1.2
47	138	1.2
48	138	1.2
43	158	1.2
49	158	1.2
49	162	1.2
49	165	1.2

Supplementary References

1. A. C. Walls *et al.*, Structure, Function, and Antigenicity of the SARS-CoV-2 Spike Glycoprotein. *Cell* **183**, 281-292 (2020).
2. Z. Zhang *et al.*, A Systematic Methodology for Defining Coarse-Grained Sites in Large Biomolecules. *Biophysical Journal* **95**, 5073-5083 (2008).
3. E. Lyman, J. Pfaendtner, G. A. Voth, Systematic Multiscale Parameterization of Heterogeneous Elastic Network Models of Proteins. *Biophysical Journal* **95**, 4183-4192 (2008).
4. L. Li, C. Li, Z. Zhang, E. Alexov, On the Dielectric “Constant” of Proteins: Smooth Dielectric Function for Macromolecular Modeling and Its Implementation in DelPhi. *Journal of Chemical Theory and Computation* **9**, 2126-2136 (2013).
5. M. S. Shell, The relative entropy is fundamental to multiscale and inverse thermodynamic problems. *The Journal of Chemical Physics* **129**, 144108 (2008).
6. A. Chaimovich, M. S. Shell, Anomalous waterlike behavior in spherically-symmetric water models optimized with the relative entropy. *Physical Chemistry Chemical Physics* **11**, 1901-1915 (2009).
7. J. M. A. Grime, J. J. Madsen, Efficient Simulation of Tunable Lipid Assemblies Across Scales and Resolutions. *arXiv:1910.05362v1* (2019).
