# Supplementary material for: Cooperative multivalent receptor binding promotes exposure of the SARS-CoV-2 fusion machinery core
Source: Nat Commun. 2022 Feb 22;13:1002. doi: 10.1038/s41467-022-28654-5 (PMC8863989; doi:10.1038/s41467-022-28654-5)
Supplement: Supplementary file 2 — Description of Additional Supplementary Files [file 41467_2022_28654_MOESM2_ESM.pdf]

Description of Additional Supplementary Files

File Name: Supplementary Movie 1
Description: Movie of S1 dissociation induced by multivalent ACE2 binding during coarse-grained molecular dynamics simulations. The two protomers within ACE2 dimers are depicted as red and blue beads, respectively, while the three S1 protomers within the spike trimer are respectively depicted as cyan, pink, and green beads. All glycans and S2 protomers are represented by grey and silver beads, respectively. Lipids are not shown for clarity.
